# Double task switching: An investigation into the effects of similarity and task-rule congruency on cognitive flexibility

**DOI:** 10.1371/journal.pone.0305675

**Published:** 2024-10-03

**Authors:** Marcel F. Hinss, Anke M. Brock, Raphaëlle N. Roy

**Affiliations:** Fédération ENAC ISAE-SUPAERO ONERA, Université de Toulouse, Toulouse, France; University of Pecs Medical School, HUNGARY

## Abstract

Similarity between tasks is an understudied factor in research on cognitive flexibility. This behavioural experiment had 31 participants perform a task switch paradigm in which participants were required to switch between 4 tasks of varying similarity. The experiment was constructed in a way that simultaneously allows for investigating the impact of mental fatigue and task-rule congruency on the participants. The results indicate that similarity between tasks substantially impacts performance with different effects on RT and accuracy. While learning effects may have negated the impact of mental fatigue across the 5 experimental blocks, a significant decrease in performance was observed within blocks. Furthermore, the exploratory analysis proposes a novel interaction between task-rule incongruent trials and the task of the previous trial. These results support the notion that neither the interference view of cognitive flexibility nor the reconfiguration view are fully adequate at explaining task switch costs if similarity is added as a factor. The presented study presents strong evidence that fundamental findings in the domain of cognitive flexibility may not map linearly to more ecological settings where tasks are often more dissimilar.

## 1 Introduction

Cognitive flexibility, the ability to adapt our behaviour and thoughts to the environment, is one of the most important aspects of human cognition [1]. A classical paradigm to study cognitive flexibility is the task switch paradigm [2–7]. This paradigm requires the participant to switch between two or more relatively simple tasks continuously. Results consistently show that switching between tasks results in task switch costs (TSC) in the form of increased reaction times (RT) and error likelihood following a switch [2–7].

The existence of TSC poses the question of how these effects occur. Two main theories explain this behavioural phenomenon. The Interference and the Reconfiguration views [8, 9]. The interference view was first proposed by Allport in 1994. The idea is that the components relevant to the previous task remain activated and interfere with the current trial if a switch occurs. In repetition trials, these components are also activated but do not interfere as they are the same for trial n-1 and trial n [8, 10]. The Reconfiguration view, on the other hand, proposes that task set parameters need to be activated or retrieved from long-term memory when a switch occurs [11].

In the pursuit of validating one or the other theories, a multitude of variables, such as preparation time, previous interference residual switch costs, working memory, bivalence, similarity, and other factors, have been investigated concerning their relation to TSC [4, 10–13]. For a review of the two opposing theories and a summary of the influences of factors on TSC see [13, 14].

The similarity factor is of interest for research on cognitive flexibility, especially as most fundamental research has only focused on very similar tasks [7, 15]. Similarity can be seen from the perspective of the task set. The task set is the procedural schema in which the mental resources must be configured to perform each task [5]. Similarity can be seen as the distance between the task-sets of two tasks. Recent results from a study by [16] show that learning in a task-switching context depends on the task set rather than the physical features or the stimulus presented. The study shows that participants can apply the experience from previous trials to a novel trial, even if the presented stimulus is unknown. By investigating similarity, we can examine whether task-set information is partially transferrable across tasks.

In many experimental designs, ecological validity is not always given, as task switching, in reality, may occur between vastly dissimilar tasks. Ecological validity is essential to cognitive flexibility, as research has shown that issues with cognitive flexibility have significant real-world implications, especially in the domain of Human-Machine Interaction (HMI; [17–21]). If the fundamental research conducted on cognitive flexibility is to be applied to real-world contexts and problems, the question arises as to what extent these fundamental findings also apply to a realistic context. If this is not the case, then it is of interest to see the differences between task switching in a fundamental context and task switching in an ecologically valid context [22–24]. This question makes similarity between tasks a critical aspect of understanding cognitive flexibility, as it could be argued that classical task switch paradigms involve tasks [1] that are more similar than task switching that is required of operators in complex operations [18].

Some studies have investigated the effects of similarity and closely related effects on task switching [15, 22–25]. In study [2], participants were presented with rectangles of different shapes and colours. Participants had to switch between two sets of two similar tasks and make binary decisions. The tasks were judgments of height, width, hue, and intensity. The assumption, which was later validated, was that the width, height, and hue and intensity tasks were more similar. The responses within that study were univalent (each response possibility for each task was mapped onto a different key on the keyboard or a different vocal reaction). However, the stimuli were bivalent, meaning that each stimulus could be applied to all tasks (e.g., the number 4 can be used for high/low tasks and even/odd tasks).

Bivalency is of interest, especially when looking at task-rule incongruent and task-rule congruent responses [13]. In studies where both the stimuli, as well as the reactions, are bivalent (the same responses are used for all keys, and all stimuli can be applied to all tasks), task-rule congruent stimuli (stimuli for which the correct response to the relevant as well as the irrelevant task are the same) are typically faster than task-rule incongruent stimuli (stimuli for which the proper response to the appropriate task is not the same as to the irrelevant task [12]. This does not directly apply to the study of [15], as here, each response was mapped to a different key, and therefore, the effect of task-rule incongruency was kept constant. The recent study [22] used a similar dimensional design of different tasks. In addition to switching between two tasks, the switch rules could also be different. The work of [24] also uses a dimensional design, but here, instead of similarity, compatibility of the stimuli is added as a second factor. Further expanding is the 2004 study by Kleinsorge [23]. Using a factorial design, participants must switch between a parity or numerical magnitude task while focusing on the numeric value or the number of digits displayed. Therefore, the participants in this study had to switch between 4 different tasks. However, except for switches between tasks where both factors were changed, the tasks all had one factor in common (either the actual task instruction or the part of the stimulus on which to focus).

Several questions arise: are the task-rule incongruency effects that occur in studies using a bivalent design (when the task-relevant cue indicates answer A and the task-irrelevant cue indicates answer B) additive? That is to say, in a task switch design using 4 different tasks, is there a difference between 1 irrelevant cue biasing towards the incorrect answer and 3 irrelevant cues biasing towards the wrong answer? What has a stronger impact on TSC, similarity, or task-rule congruency? Does it matter if the task-rule incongruent information comes from a similar or dissimilar task?

Beyond exploring these questions that have a fundamental interest in understanding cognitive flexibility, there is also a motivation for more ecological questions. Task switching in an ecological context may involve switching between tasks where similarity and bivalence are independent. The proposed study also lacks ecological validity. However, investigating the mechanisms of cognitive flexibility in the context of more or less dissimilar tasks may facilitate future research to experiment with vastly dissimilar tasks that better resemble the real world. To pay attention to factors vital to ecological validity, mental fatigue was identified as an essential covariate. Mental fatigue, as defined by the APA is:

*“a decline in performance on a prolonged or demanding research task generally attributed to the participant becoming tired or bored. The fatigue effect is an important consideration when administering a lengthy survey or test in which participants’ performance may worsen simply due to the challenges of an extended task.”* [26]

Many studies with a multitude of different tasks reliably show an increase in RT and a decrease in accuracy (ACC) as mental fatigue increases [27–29]. In many of these studies, mental fatigue is approximated with Time-on-Task (TOT). Studies on cognitive flexibility show that the increase in TSC is significantly bigger than the overall increase in RT and decrease in ACC [30–33]. Furthermore, mental fatigue has been identified as a risk factor in several types of complex operations that simultaneously require the operator to maintain a degree of cognitive flexibility [21, 34–36]. Beyond complex operations, deficits in cognitive flexibility are also associated with several psychiatric/psychological conditions. Research has shown deficits in cognitive flexibility related to Parkinson’s disease, Schizophrenia, attention deficit/Hyperactivity Disorder (ADHD), and Depression [37, 38]. The results of this study may also help understand the impact the pathologies have on the lives of those affected, in the context of cognitive flexibility.

*NOTE: For the remainder of this article, we will no longer use the terms bivalency and univalency for the sake of simplicity. In the experimental design, 7 different levels of task-rule congruency were implemented*.

- *Trials in which only the relevant information for the given task is presented (univalent) are **task-rule neutral trials***.
- *Trials in which additional information is presented, but the information is in line with the correct response (bivalent) are **task-rule congruent trials***.
- *Trials in which additional information does not align with the correct response (bivalent) are **task-rule incongruent trials***.

### 1.1 Hypotheses

1. Task Switch Costs:
  (a) Following a task switch, the RT on said trial is higher than the average RT on trials of the same task where previously no switch occurred.
  (b) Following a switch of the task, ACC on said trial is lower than the average ACC on trials of the same task where previously no switch occurred.
2. Similarity Effects:
  (a) Task Switch Costs (hypothesis 1; RT and ACC), are more pronounced if the switch is between two more dissimilar tasks
3. Task-Rule Congruency Effects:
  (a) A task-rule incongruent stimulus results in a higher RT regardless of whether the trial is a switch trial or a repetition trial.
  (b) A task-rule incongruent stimulus results in a lower ACC regardless of whether the trial is a switch trial or a repetition trial.
4. Mental Fatigue:
  (a) As TOT increases, the RT increases regardless of whether the trials are switch trials or repetition trials.
  (b) As time on task increases, error likelihood increases regardless of whether the trials are switch trials or repetition trials.
  (c) As TOT increases, Task Switch Costs (hypothesis 1) increase.

## 2 Materials and methods

### 2.1 Ethics statement

Ethical consent was obtained from the Comité d’Ethique de la Recherche—CER at the Université de Toulouse (ID 2022–521) on the 2nd of June 2022. Data collection occurred from January until April of 2023.

### 2.2 Design

The DTS protocol consists of four subtasks: two based on the numeric value of the displayed stimuli and two based on their visual appearance. The numeric subtasks consist of the LOW/HIGH and EVEN/ODD tasks. For the LOW/HIGH task, participants must indicate, using a keystroke, whether a single number is higher or lower than 5. The number 5 is never present in the task, and all LOW/HIGH task stimuli are on the left. In the EVEN/ODD task, participants must decide if a stimulus (again, a single number, but located on the right side) is even or odd. Again, participants are instructed to respond with a keystroke. For the visual tasks, participants have to either perform the COLD/HOT task, where they have to decide whether the stimulus is presented in either a hot (red/orange hue) or a cold colour (blue/green hue), or they have to perform the VERTICAL/HORIZONTAL task. Here, a pattern’s orientation must be determined (see Fig 3 for an example array of stimuli for each condition). Participants must perform consecutive trials, where the task and stimuli change quasi-independently from trial to trial. For each trial, participants are presented with 3 quasi-independent pieces of information. They are presented with an instruction for the current trial in the form of the task name (e.g. VERTICAL/HORIZONTAL) 300 ms before the actual stimulus. Two stimuli then follow the instructions as signs or numbers that may vary in value, pattern and colour. Based on the stimuli and the instruction, participants are instructed to respond as fast as possible, using either the S or the L key on a keyboard (keys were selected as they are located at a comfortable distance on the keyboard to avoid straining the hands of the participants).

There are 168 different conditions for each trial (4 Tasks * 3 Switch conditions * 7 Task-Rule Congruency Conditions * 2 Response types). However, as differences in the performance of individual tasks and response types are not hypothesized to impact participants’ performance, 21 main conditions (3 Switch conditions * 7 Task-Rule Congruency Conditions) remain.

#### 2.2.1 Task switch conditions

**Fig 1:**
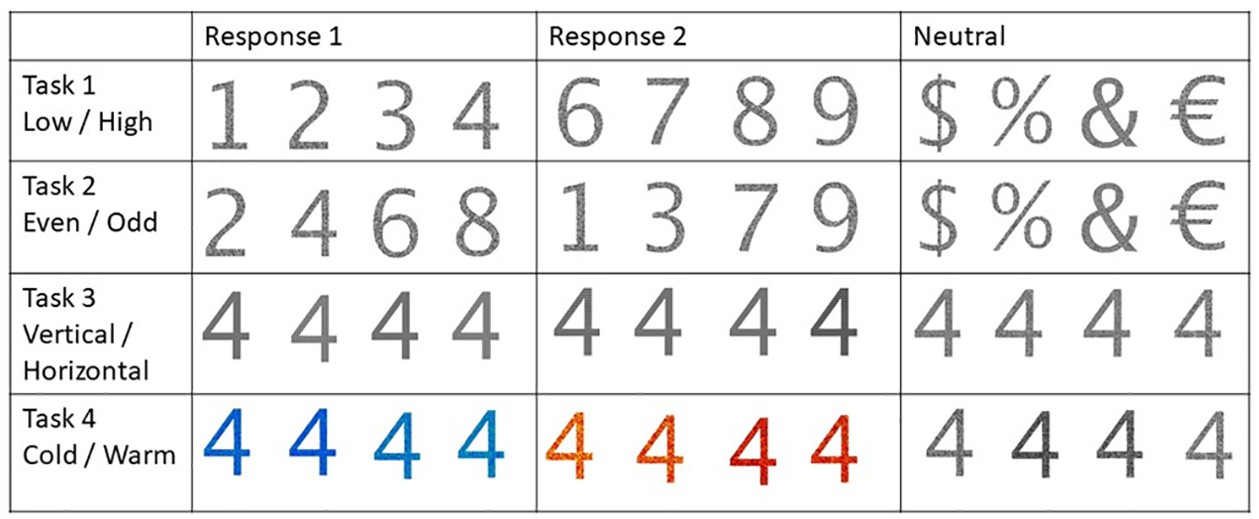
Examples of different stimuli for all subtasks of the task. Note: Task 3 Horizontal and Vertical may be difficult to see in this picture. Please refer to Fig 2.

**Fig 2:**
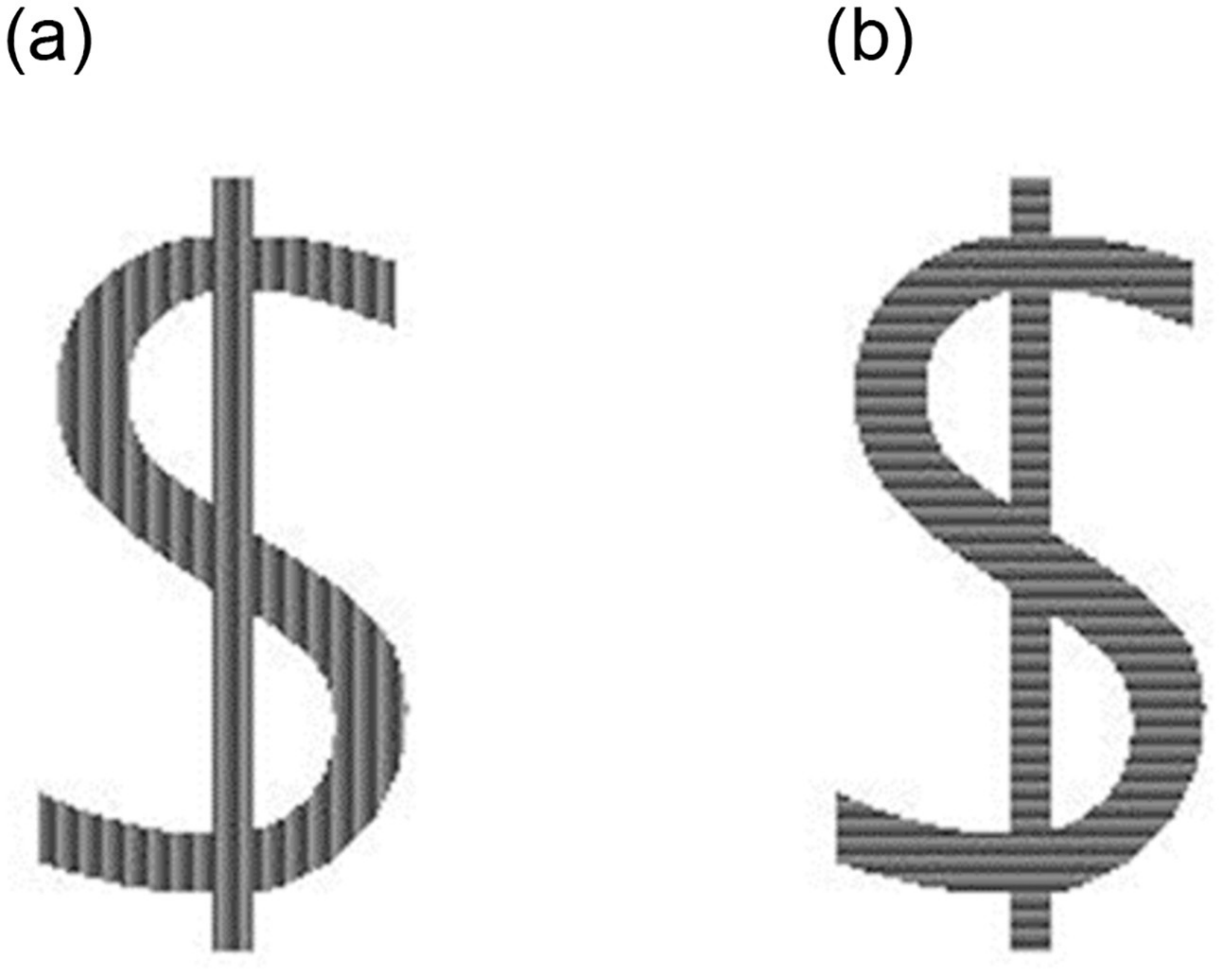
Larger example of stimuli with cue relevant for Task 3. Left: vertical orientation. Right: horizontal orientation.

Each trial (n) is classified into one of the three Task Switch Conditions depending on the task in trial n-1. If the task remains the same, the trial is a repeat trial. Suppose the task switches but remains within the same domain (Numeric: LOW/HIGH to EVEN/ODD or Visual: VERTICAL/HORIZONTAL to COLD/HOT and vice versa). In that case, it is considered an internal switch trial. Finally, switches from one domain into the other (LOW/HIGH or EVEN/ODD to either VERTICAL/ HORIZONTAL or COLD/HOT and vice versa) are considered external switch trials (Figs 1 & 2).

#### 2.2.2 Task-rule congruency conditions

**Fig 3:**
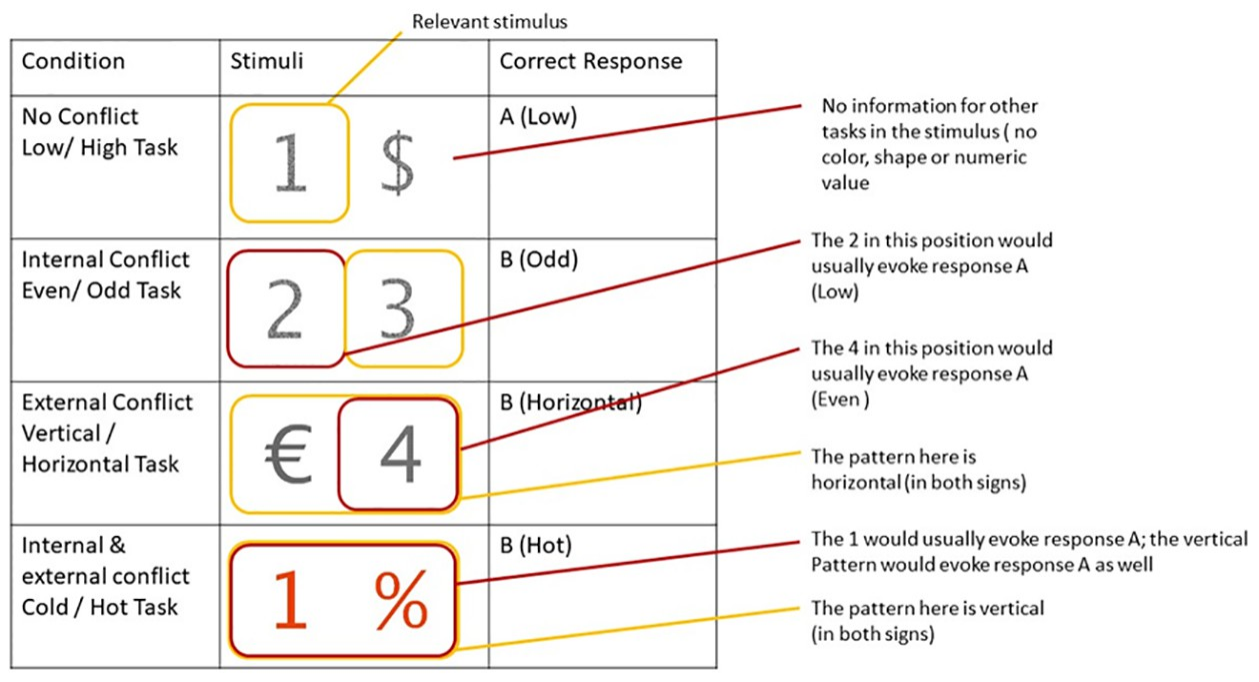
Examples of the different task-rule congruency conditions.

Depending on the presented stimuli, a trial may be more or less task-rule congruent. This occurs when the task-relevant information is supplemented by task-irrelevant information that is biased towards the correct or the incorrect answer. 7 different conditions exist. In the task-rule neutral condition, the stimuli only present one piece of information, the relevant one. In Fig 3, the stimuli in the task-rule neutral condition provide no information pertinent to each of the 3 non-relevant tasks.

The *internal task-rule incongruent condition* adds information indicating the opposite response (E.g. clicking L instead of S). This information is relevant for the other task of the same domain (the similar task). The information is therefore *internal* (same domain) and *task-rule incongruent* (bias towards the wrong response). In the example, the participant has to perform the Even/ Odd task, which is always based on the right number. Therefore, 3 is the relevant number; the correct answer would be a click on the letter ‘L’. The other stimulus (the number 2) is relevant only to the high/ low task and would click on the letter’ S’ if the participant were performing said task.

The *external task-rule incongruent condition* is similar; the only difference is that the non-relevant task is not from the same domain but is one (and always only one) of the tasks from the other domain.

The *double task-rule incongruent condition* combines the two previous conditions, where the other internal task and one (and always only one) of the external tasks bias the participant towards an incorrect response.

Three more conditions were added to the experiment. These mirror the task-rule incongruent conditions. However, the task-irrelevant information is congruent with the task at hand [9]. In the *external task-rule congruent condition*, this would, for example, mean that a participant would be instructed to do the Low/High task and see a ‘3’ as a left stimulus in a blue hue. The value 3 would mean that the number is low (corresponding to the letter ‘S’ on the keyboard), and the colour is cold (also corresponding to the letter ‘S’ on the keyboard).

Added stimuli will always be either task-rule congruent or task-rule incongruent.

During task-rule congruent trials, it is impossible to determine if a participant responded correctly due to the relevant task or the irrelevant information. Nevertheless, the inclusion of these conditions was necessary to be able to study the effect of congruency.

The locations of the relevant stimuli for the numeric tasks were chosen to be Low/High on the left and Even/Odd on the right for the following reasons. Should participants perceive two single digits not as, e.g. seven and one but as seventy-one, this would not change the responses. As the Even/Odd relevant number is located (right or second), it determines whether a number is odd or even, independent from the number to the left of it (that is to say, both 1 and 71 are odd numbers). Similarly, the size of the first number is somewhat independent of the second number (7 is larger than 5, but 71 is also larger than any number in the fifties).

#### 2.2.3 Trial setup

**Fig 4:**
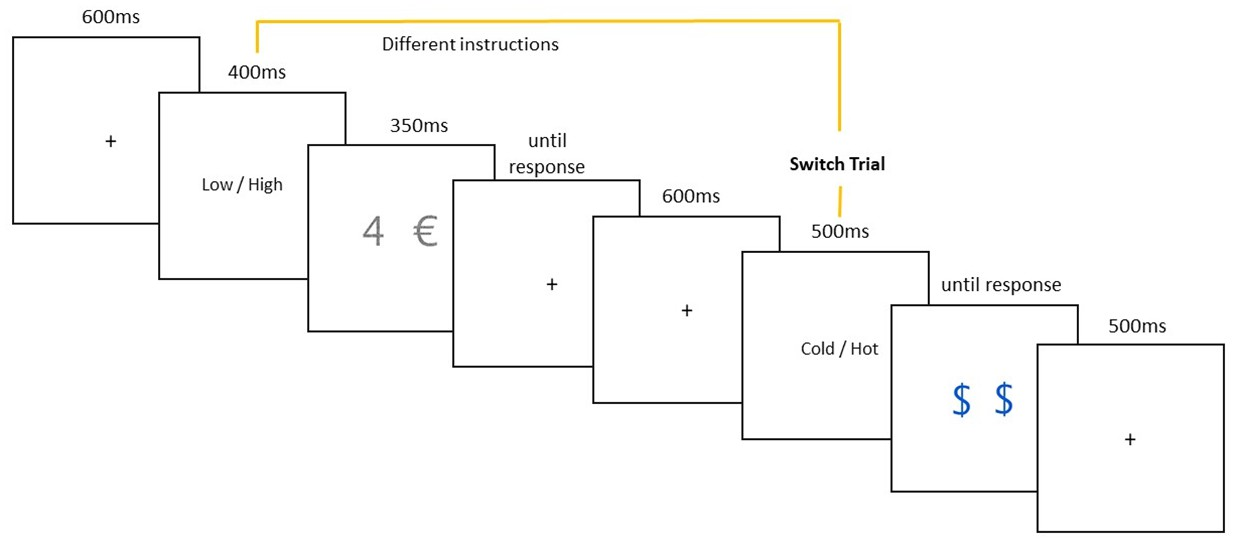
Structure of a trial.

Each trial begins with a fixation cross in the middle of the screen for 350–850 ms (600 ms with a 250 ms jitter). This is followed by trial instructions presented for 400 ms. These instructions give participants the current tusk in the form of the task’s name (e.g. LOW/HIGH). Following a 300ms delay, participants are presented with the stimulus (350 ms), where they can respond with a keystroke. If participants take longer than 350 ms to respond, the stimulus disappears. Once a response is recorded, a new trial begins (See Fig 4). The task was computed using PsychToolBox-3 (http://psychtoolbox.org/) in Matlab and is presented on a desktop computer. Participants are not randomized (within-subjects design). However, the trials that participants will complete are quasi-random. That is to say, trials will appear in a random order, which ensures a balanced design and equal probabilities of each trial occurring.

### 2.3 Sample size

**Fig 5:**
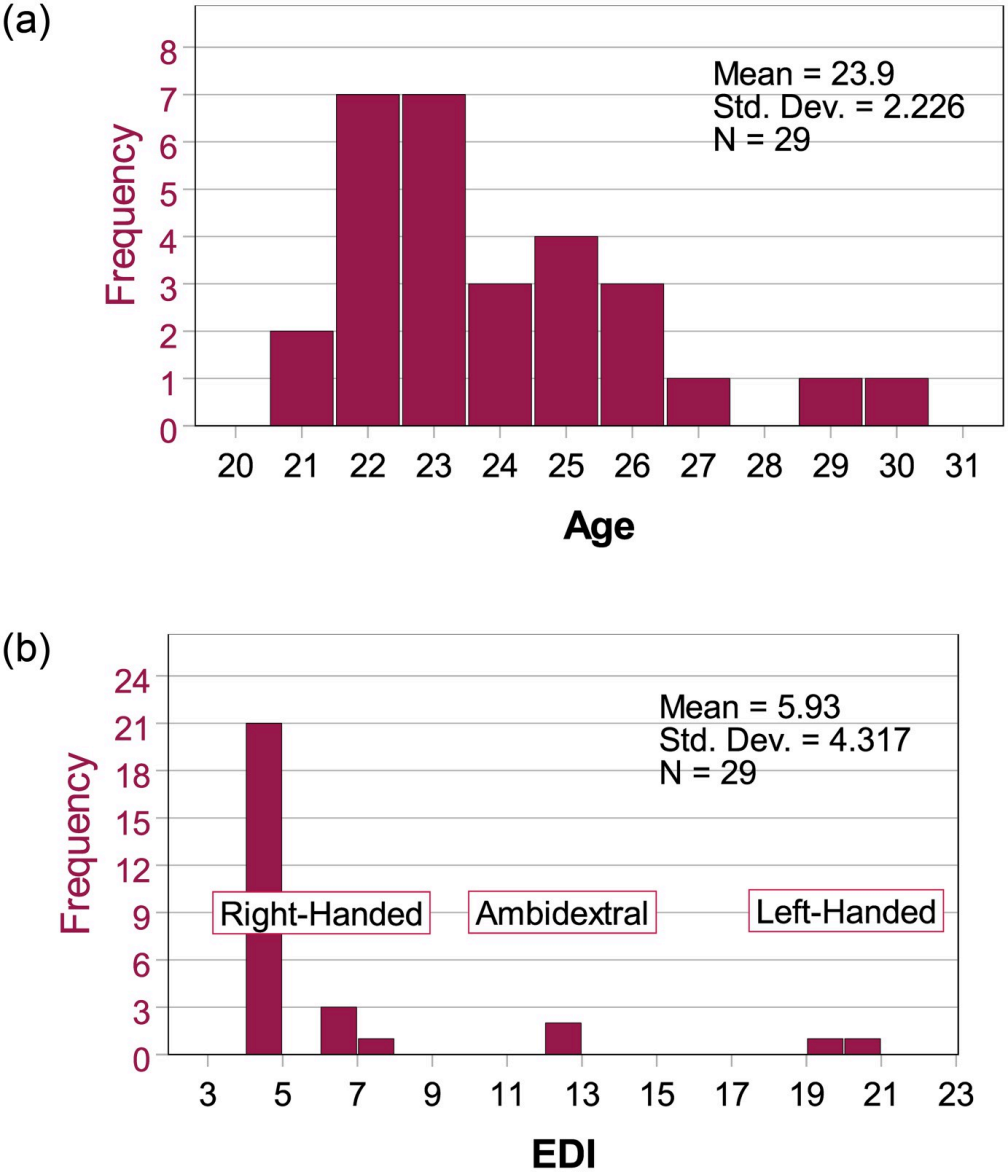
Summary of demographics: Left: Distribution of age; Right: Distribution of handedness as assessed by the EDI [40].

The approximate range of required participants was determined using GPower [39]. Based on the reported statistics [3, 15], their reported effect sizes ranged from 0.84 to 1.81 (f-squared). For a one-tailed test with an alpha of 0.05, the sample size required ranges from 15 to 25 participants. These numbers are only approximations, as the effects we intend to observe are not precisely the same as the ones reported in the aforementioned studies. From 31 Participants that were recruited, 2 participants had to be discarded for non-completion of the study. Hence, the final sample comprised 29 participants (17 females, mean age 23.9 years). The scores on the Edinburgh Handedness Questionnaire (EDI) range from 4 (right-handed) to 20 (left-handed). 25 participants had EDI scores indicating right-handedness (5–10), with three participants scoring as ambidextral (11–19) and one participant as left-handed (20–25). A visual representation of the demographics can be found in Fig 5.

### 2.4 Recruitment inclusion criteria and compensation

Participants were recruited by e-mail and via paper announcements in the institute buildings at ISAE-SUPAERO. Participants had to be between 18 and 30 years old, have obtained a level of study baccalaureate minimum, have normal or corrected-to-normal vision and hearing, be affiliated with social security, and have signed an informed consent form. Participants were prohibited from participating if they met any of the following criteria: Protected persons, presence of known neuropsychological disorders, significant visual or hearing impairment, current treatment with psychotropic medication or substances, positive g-test or being a nursing woman. In addition, due to the nature of the experiment, colourblind people were excluded from participation. As recommended to minimize the risk of an invasion of privacy, all criteria (inclusion and/or exclusion) were listed together in the inclusion criteria sheet, and potential participants were asked whether they met all the criteria rather than asking them whether they met each criterion one by one. Due to the nature of the experiment, participants were also tested for colourblindness before signing the inclusion/exclusion criteria. To do so, the Ishihara test for colourblindness was employed [41]. Participants were compensated in gift vouchers (Illicado, illicado.com). Upon completion, participants received vouchers worth 20€ (8€ for the first training session and 12€ for the more extended testing session). Participants were informed that dropping out after the first session would not impact their right to receive the 8€ voucher.

### 2.5 Procedure

After the participant’s arrival for the first session, the participant was asked to complete the Ishihara colourblindness test before filling in the informed consent and information sheets. The participants were told they were permitted to ask questions at any time and were also asked in what language they would like to perform the task (French or English). They then filled in the Demographics questionnaire, the EDI, St Mary’s Hospital Sleep Questionnaire (SQN) and a KSS and the Samn-Perelli Fatigue (SPF) 7-point Likert scale [42]. Next, the participants were familiarized with the DTS protocol and performed a training session until they acquired adequate skills. For the training session, participants performed at least 5 blocks (96 trials each, 3,5 minutes without feedback, 4 minutes with feedback) of the DTS protocol. During the first and second blocks, participants received feedback following each trial to increase their learning rates. Subsequent blocks excluded the feedback to mimic the experimental conditions completely. Additional training blocks were added for some participants, ensuring adequate task performance. Additional training was added if the last completed block of trials showed significant improvement in RT over the preceding block. Extra blocks were only added with the explicit consent of the participant. After the training session, the participants filled in a KSS as well as an SPS scale (see S5 and S6 Figs).

The second session was scheduled no longer than 14 days after the first session. A second session was required to observe the effects of mental fatigue. This is necessary because if the entire experiment were performed in one session instead of two, we would expect participants to be slightly tired at the onset of the main task following the long training period. Would we reduce the length of the training period to reduce this effect, due to the complexity of the task, this might result in strong learning effects during the main experimental task.

**Fig 6:**
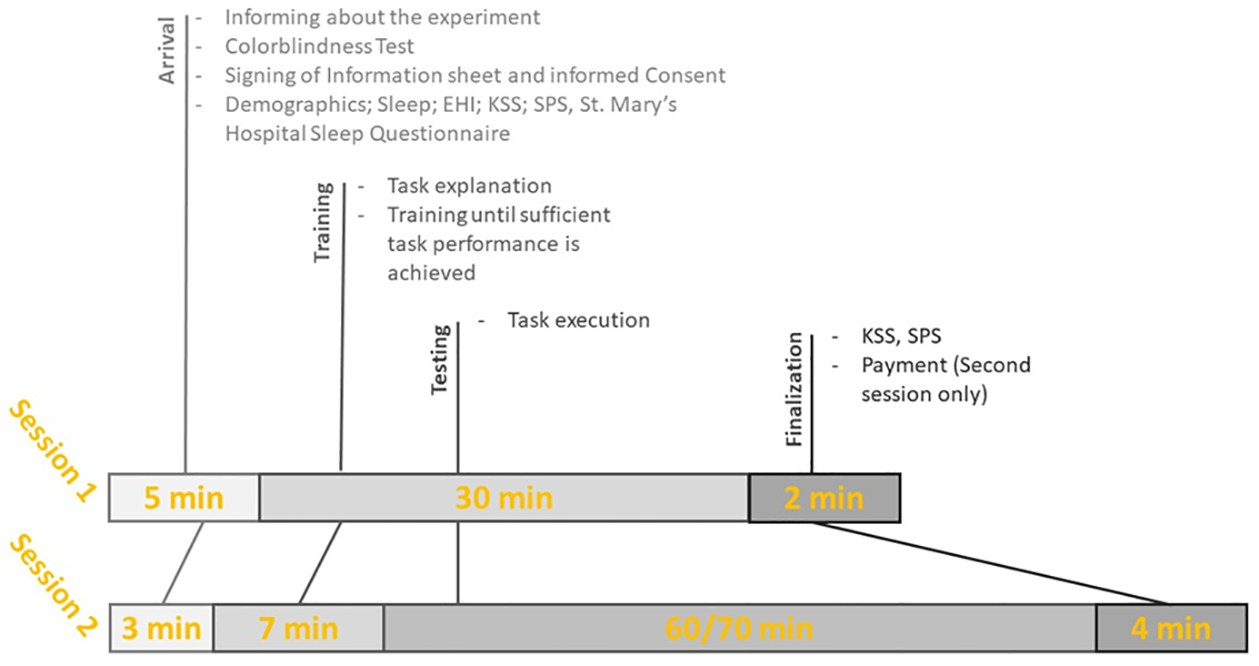
Schema of the experimental procedure.

The procedure of the second session was mostly similar to that of the first session. After filling in an SQN, participants performed a much shorter second training session intended to help the participants remember the task. Participants completed half a training block of trials (48 trials) with feedback and another half without feedback. Next, participants filled in a KSS and an SPS scale and performed the experimental DTS protocol as detailed above. Participants completed 5 Blocks of 336 trials each. In between blocks, participants had a short break of 28 seconds, as proposed by [43], to avoid recovery from the TOT effects. Upon completing the task, participants finished the experiment by responding to a KSS and an SPS scale, before being thanked for their participation and paid (See Fig 6).

### 2.6 Variables

#### 2.6.1 Independent variables

The following stimuli were used in this study:

- Numeric Value: The value of the presented stimuli was manipulated. Participants saw combinations of the numbers 1,2,3,4,6,7,8 and 9 and symbols %, &, € and $.
- Color: The stimuli colour was manipulated to either be hot (red/orange hues), cold (blue/ green hues) or neutral (grey hues).
- Pattern: The pattern of the stimuli was manipulated and could either show a horizontally oriented pattern, a vertically oriented pattern, or a random pattern.
- Task: The task participants had to complete given the stimuli could be adapted, as seen in Fig 1. The four tasks were Low/High, Even/Odd, Cold/Hot, and Vertical/Horizontal.
- Task-rule congruency: Depending on the stimuli, the experiment manipulated the degree of task-rule congruent/incongruent information presented. See Fig 3.

The following covariate was considered: TOT, *i.e*. how long the participant has been performing the task, was incorporated into the analysis as an indicator of mental fatigue in the form of the factor blocks.

#### 2.6.2 Dependent variables

The following measures were recorded:

- ACC: The number of correct answers during the task.
- RT: The RT on each trial. RT here was measured as the time from stimulus presentation to response (keystroke).
- Subjective Questionnaire Data: The data from several subjective questionnaires. All questionnaires were presented to the participants in a paperless digitalized form.

### 2.7 Questionnaires

#### 2.7.1 Demographics questionnaire

The demographics questionnaire included questions assessing age, gender and occupation. This questionnaire can be found in the S1 Fig.

#### 2.7.2 Edinburgh Handedness Inventory

The shortened version of the Edinburgh Handedness Inventory encompasses 4 items (e.g. writing) scored on a 5-point scale ranging from:” Always right; Usually right; Both equally; Usually left; Always left”. This shortened version is a faster measure while maintaining its reliability [40]. A copy of the inventory is attached in the S2 Fig.

#### 2.7.3 Karolinska Sleepiness Scale (KSS)

The KSS was developed to measure subjective sleepiness at any given time. It is a simple 9-point scale ranging from “extremely 1 = alert” to “9 = extremely sleepy–fighting sleep”. The scale has been validated and used frequently and provides a simple and fast measure that provides a reliable result [44]. The scale is in the Appendix in the S6 Fig.

#### 2.7.4 Samn-Perelli Fatigue (SPS)

The estimation of fatigue was performed using the Samn-Perelli Fatigue scale (SPF; [45]). This scale consists of 7 points and is used in aviation to assess crew fatigue. The French version comes from a report by the International Civil Aviation Organization [41]) but has not been psychometrically validated (S5 Fig).

#### 2.7.5 Ishihara colorblindness test

The Ishihara test for colourblindness [41] encompasses stimuli that can determine the degree and type of colourblindness a person has. It was presented in printed form, using a validated version of the test made available by the US Department of Infectious Diseases (S3 Fig). Participants were informed that a licensed physician is required for a medically accurate test. Should the suspicion of colourblindness arise during the assessment, participants were notified and advised to seek a medical professional.

#### 2.7.6 The St Mary’s Hospital Sleep Questionnaire (QSN)

The QSN was used to estimate the sleep quality on the previous night. A translated version of this questionnaire has been produced (S4 Fig) [46]. The scoring of this questionnaire is not standardized due to the use of Likert scales and free responses. In addition, two questions regarding caffeine consumption have been added to this questionnaire.

### 2.8 Analysis plan

The general inference criterion is a p-value of *p* < .05. In multiple comparisons, we adjusted that criterion according to the adjusted Bonferroni method. Assumptions for each statistical test were checked and accounted for if not satisfied.

#### 2.8.1 Outliers

Two steps were employed to detect and reject outliers. First, trials with RTs above 5 seconds were rejected and counted as misses. In addition, an outlier detection was performed based on the interquartile range criterion. Trials with RT outside the 1.5x criterion were excluded This was done for trials grouped by condition.

#### 2.8.2 Primary analysis

The analysis was performed using a Mixed (Multi-Level) Regression, as this allows for accounting for the effects of participants and tasks. A first mixed multilevel regression was performed on the dependent variable of RT (in ms). The independent variables were Trialtype (3 Levels), Task (4 Levels), Block (5 Levels) and Task-Rule Congruency (7 Levels). The dependent variable was RT (in ms).

In a second Mixed multilevel logistic regression, ACC was analyzed. The independent variables were Trialtype (3 Levels), Task (4 Levels), Block (5 Levels) and Task-Rule Congruency (7 Levels). The dependent variable was ACC (Binary). To account for participants and task effects, both were dummy-coded and included as IV effects in the Mixed Regression for both analyses. The analysis was first performed with a random intercept + Autoregressive Moving Average (ARMA)/ Autoregressive of Order 1 (AR1) to determine the most fitting covariance structure. Random slopes were added if the fixed model was reduced or followed a test for slope variance of the IV effects.

Expected Results: The expected results based on the hypotheses are detailed in Table 1.

**Table 1:** Summary of the expected results with corresponding hypotheses.

Analysis Group	Hypothesis	Expected effect
**Task-Switch Costs**	Following a switch of the task, RT on said trial is higher than the average RT on trials of the same task where previously no switch occurred	The effect of trial-type (switch vs repeat) is significant in the Mixed Multilevel Regression (higher RT for switches)
	Following a switch of the task, ACC on said trial is lower than the average ACC on trials of the same task where previously no switch occurred.	The effect of trial-type (switch vs repeat) is significant in the Mixed Multilevel Logistic Regression (lower ACC for switches)
**Similarity effects**	For all switch trials, RT will be longer if the switch occurs between dissimilar tasks	The interaction effect between trial-type (switch vs repeat) and similarity is significant in the Mixed Multilevel Regression (higher RT for dissimilar switches)
	For all switch trials, ACC will be lower if the switch occurs between dissimilar tasks	The interaction effect between trial-type (switch vs repeat) and similarity is significant in the Mixed Multilevel Logistic Regression (lower ACC for dissimilar switches)
**Task-Rule Congruency effects**	A task-rule incongruent stimulus will result in a higher RT regardless of whether the trial is a switch trial or a repetition trial.	The effect of task-rule congruency (task-rule congruent vs task-rule incongruent) is significant in the Mixed Multilevel Regression (higher RT for incongruent trials)
	A task-rule incongruent stimulus will result in a lower ACC regardless of whether the trial is a switch trial or a repetition trial.	The effect of trial-type (task-rule congruent vs task-rule incongruent) is significant in the Mixed Multilevel Logistic Regression (lower ACC for task-rule incongruent trials)
**Mental fatigue**	As TOT increases, the RTs will increase regardless of whether the trials are switch trials or repetition trials.	The covariate of TOT is significant in the Mixed Multilevel Regression (higher RT over time)
	As TOT increases, error likelihood will increase regardless of whether the trials are switch trials or repetition trials.	The covariate of TOT is significant in the Mixed Multilevel Logistic Regression (lower ACC over time)
	Following a switch of the task, RT on said trial is higher than the average RT on trials of the same task where previously no switch occurred	The interaction effect between trial-type (switch vs repeat) and TOT is significant in the Mixed Multilevel Regression (higher RT for switches as TOT increases)
	Following a switch of the task, ACC on said trial is lower than the average ACC on trials of the same task where previously no switch occurred.	The interaction effect between trial-type (switch vs repeat) and TOT is significant in the Mixed Multilevel Logistic Regression (lower ACC for switches as TOT increases)

#### 2.8.3 Exploratory analysis—Rebound effects

Rebound effects, the increase in error probability and RT following an error, are well-studied phenomena [47]. In the exploratory analysis, we investigated this using a Kruskal-Wallis one-way analysis to account for unequal sample sizes. The dependent variables were RT and ACC.

#### 2.8.4 Exploratory analysis—Task-rule congruency convergence

We were interested in whether task-rule incongruent trials were influenced by the fact that the incongruent information displayed was from the task from which the participants had just switched. For example, during trial n-1, the participant was performing the high/low task and was now instructed to switch to the horizontal/vertical task. Is there a significant difference in behaviour if the task-rule incongruent information presented in trial n corresponds to the task of trial n-1 (in our example, high/low)? To investigate this, we limited the sample to switch trials and compared trials where this occurred to all other types of trials. Four possible scenarios could occur:

1. Internal switch trial with internal task-rule incongruent information
2. Internal switch trial with double task-rule task-rule
3. External switch trial with external task-rule incongruent information
4. External switch trial with double task-rule incongruent information

A Kruskal-Wallis one-way analysis was performed to account for unequal sample sizes. The dependent variables were RT and ACC. These trials were compared to the most similar subset: internal and external switches with task-rule incongruent information presented simultaneously.

#### 2.8.5 Exploratory analysis—Timepoint mixed model

Initially, it was planned to include the trial number in the mixed model analyses on RT and ACC. However, the number of parameters to estimate by SPSS would have been computationally impossible, resulting in a singular fit. We recalculated the primary analyses with the additional factor timepoint to mitigate this. Timepoint was a division of each block into three separate points. This calculation was performed on both dependent variables ACC and RT.

#### 2.8.6 Exploratory analysis—Simple task-rule congruency models

In total 7 different conditions of task-rule congruency were explored. In a simplified analysis, we recalculated the same models but regrouped these 7 types of task-rule congruency into 3: Neutral trials, Task-rule congruent trials, and Task-Rule incongruent trials. This calculation was performed on both dependent variables ACC and RT. The sample size was unequal because the number of neutral trials was significantly lower than the task-rule congruent or incongruent trials.

#### 2.8.7 Exploratory analysis—Questionnaires

To explore possible relations between the subjective questionnaires and the dependent variables, we correlated the SPS and KSS scores with RT and ACC. Correlations with Age and EDI scores were excluded due two skewed data (young population and very few left-handed participants). We also performed a Mann-Whitney U test on both dependent variables to explore possible gender effects. Furthermore, we performed repeated measures of ANOVA with Timepoint and Session on both the KSS and SPS scores.

## 3 Results

### 3.1 Outliers

From the initial 48720 trials, 52 were removed as the RT of the parFticipants was above 5 seconds. This pertains to 0.11% of the data. Then, using the 1.5 interquartile range criterion, 7.22% further per cent of the data were removed. These data were distributed across all types of trials, therefore not biasing the analysis in any direction.

### 3.2 Reaction time

The mixed regression, with task-rule congruency, block, task, and switch, showed several exciting results. The covariance structure chosen for the analysis was AR1, as it presented a significantly better model than the more simple ID (*X*^2^(1) > 25, *p* > .001). All main and two-way interaction effects were significant (Switch: *F*(44531, 2) = 1432.98, *p* < .001, Task: *F*(43428, 3) = 1634.11, *p* < .001, Task-rule congruency: *F*(43871, 6) = 21.81, *p* < .001, Block: *F*(12425, 4) = 174.41, *p* < .001, Switch*Task: *F*(44455, 6) = 109.04, *p* < .001, Switch*Congruency: *F*(43461, 12) = 5.54, *p* < .001, Switch*Block: *F*(44419, 8) = 5.2, *p* < .001, Task*Task-rule congruency: *F*(43589, 12) = 9.42, *p* < .001, Task*Block: *F*(44335, 12) = 8.67, *p* < .001, Task-rule congruency*Block: *F*(43731, 24) = 4.18, *p* < .001).

#### 3.2.1 Switches

**Fig 7:**
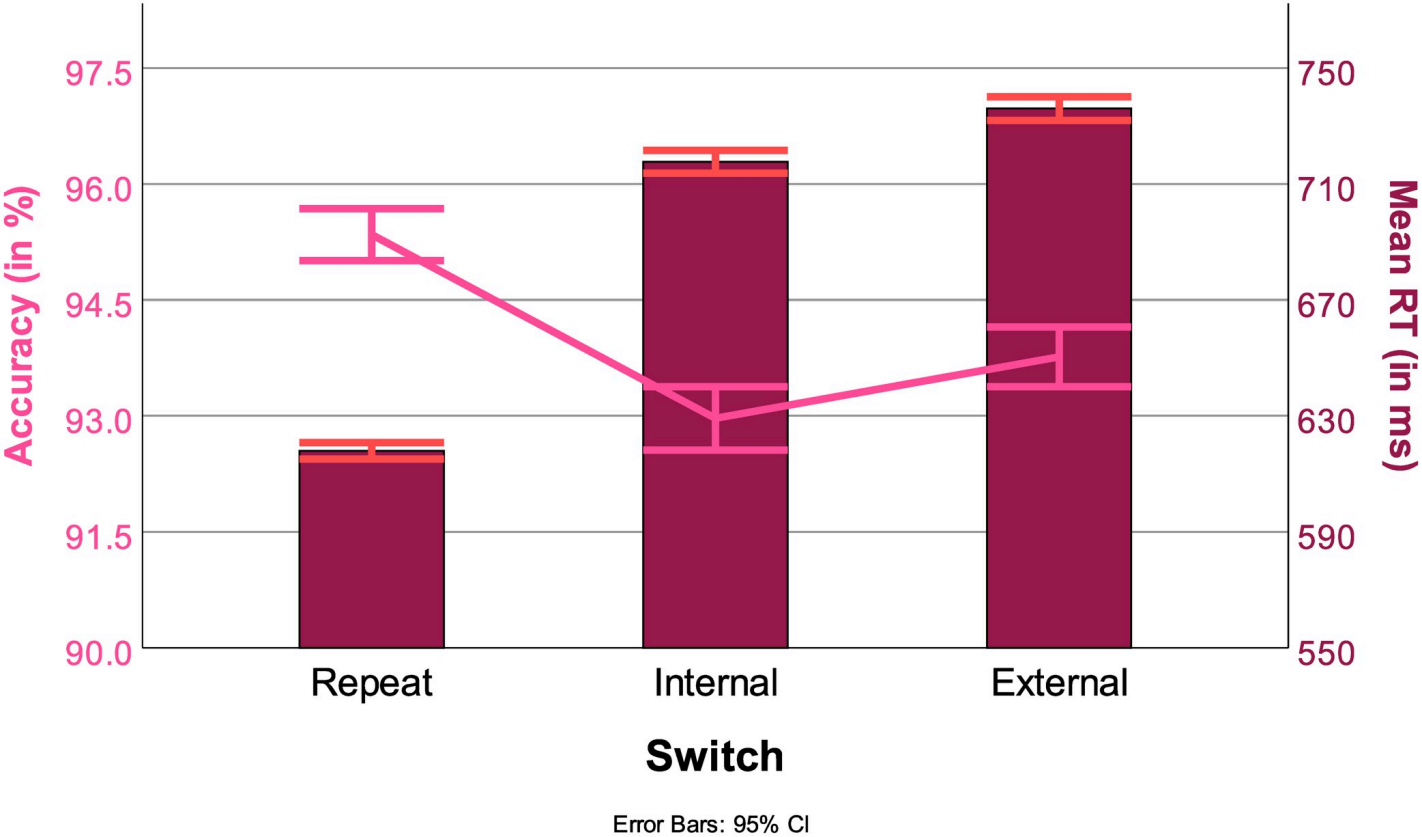
Effect of the type of switch trial on performance. Bars: RT (in ms); Lines: ACC (in %), with 95% confidence intervals.

As expected, repetition trials had the fastest RTs with 624 ms (std err: 12) (Repeat vs Internal switch: *t*(43996), *p* < .001, Repeat vs External switch: *t*(45030), *p* < .001). Internal switch trials were also significantly faster (723 ms, std err: 12) than External switch trials (740 ms, std err: 12) (Internal vs External switch: *t*(45118), *p* < .001). RTs across the types of switches are represented by the bars in Fig 7.

#### 3.2.2 Tasks

**Fig 8:**
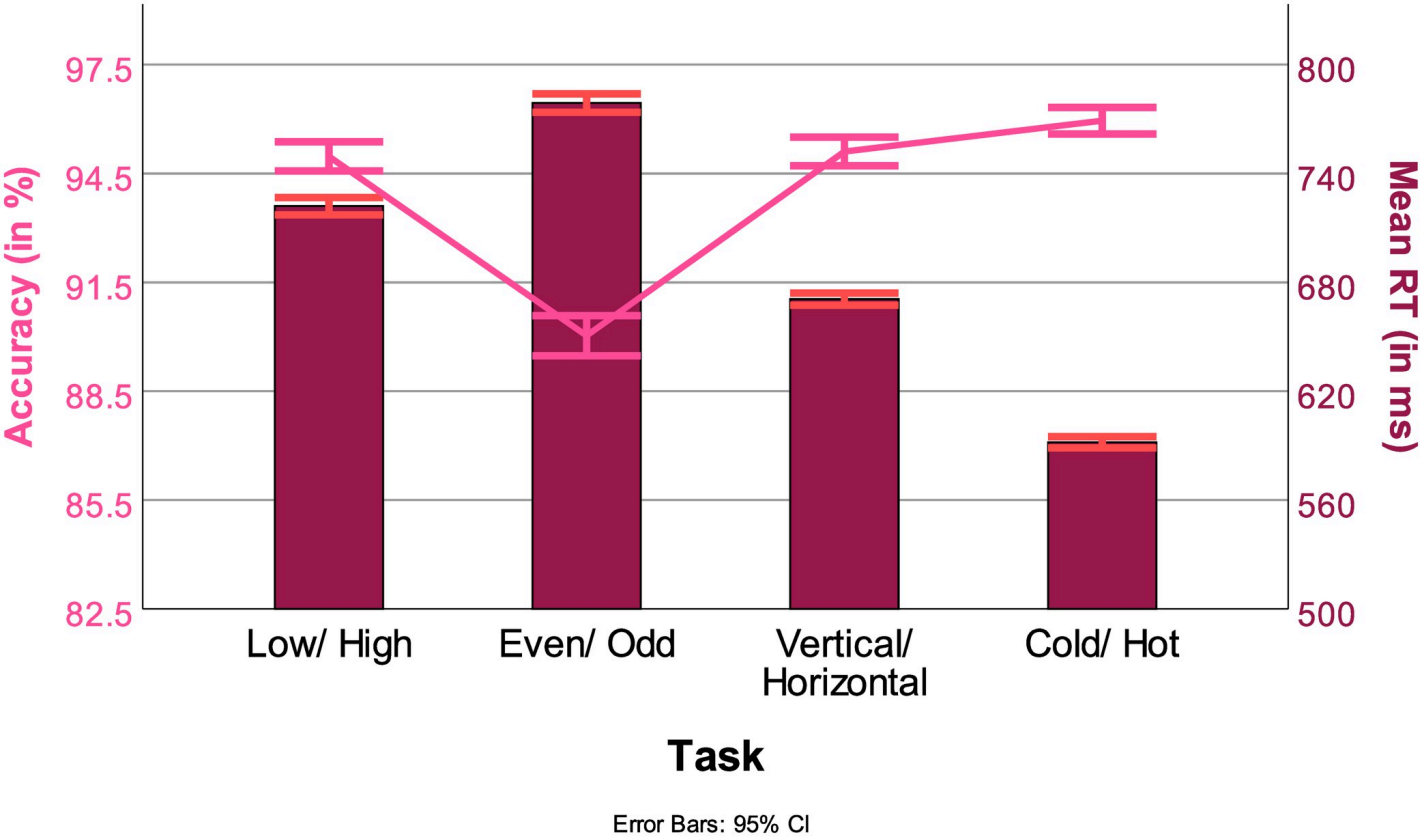
RTs (in ms, bars) and ACC (in %, line) across all tasks, with 95% confidence interval.

In terms of RT, all tasks differed from each other significantly. Participants were the fastest in the visual tasks (Cold/Hot 596 ms, Vertical/ Horizontal 675 ms) as compared to the numeric tasks (Low/High 728 ms, Even/Odd 784 ms) (Low/High vs Even/ Odd *t*(45026), *p* < .001, Low/High vs Vertical/Horizontal *t*(42438), *p* < .001, Low/High vs Cold/Hot *t*(42356), *p* < .001, Even/Odd vs Vertical/Horizontal *t*(42660), *p* < .001, Even/ Odd vs Cold/Hot *t*(42553), *p* < .001, Vertical/ Horizontal *t*(44843), *p* < .001). The bars in Fig 8 represent RTs across tasks.

#### 3.2.3 Block

**Fig 9:**
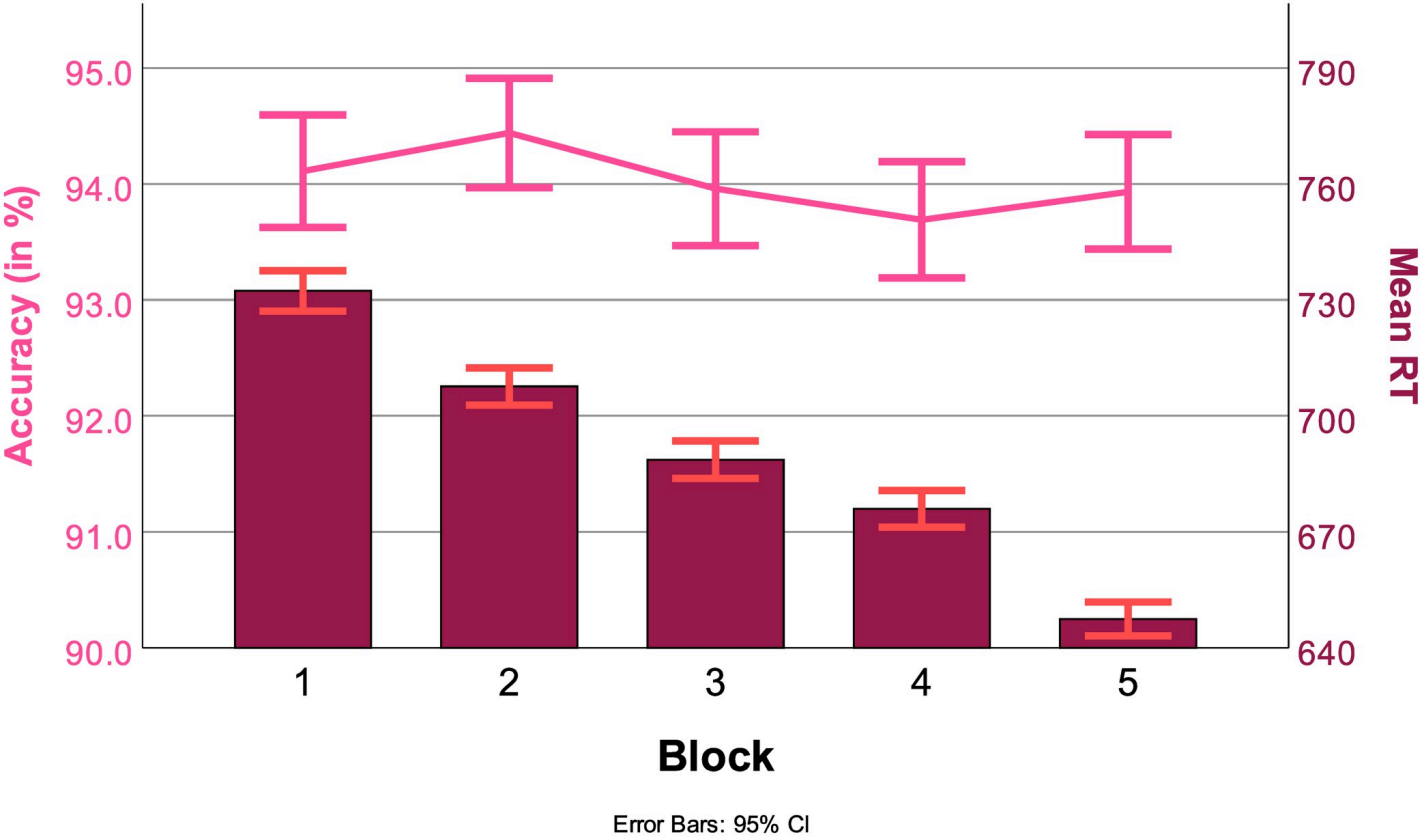
RTs (in ms, bars) and ACC (in%, line) across blocks, with 95% confidence interval.

Across blocks, RT decreased from 739 ms (Std. Err = 13) to 653 ms (Std. Err = 12). The pairwise comparisons between all blocks were significant at a p-value of *p* > .01. RTs across blocks are represented by the bars in Fig 9.

#### 3.2.4 Task-rule congruency

**Fig 10:**
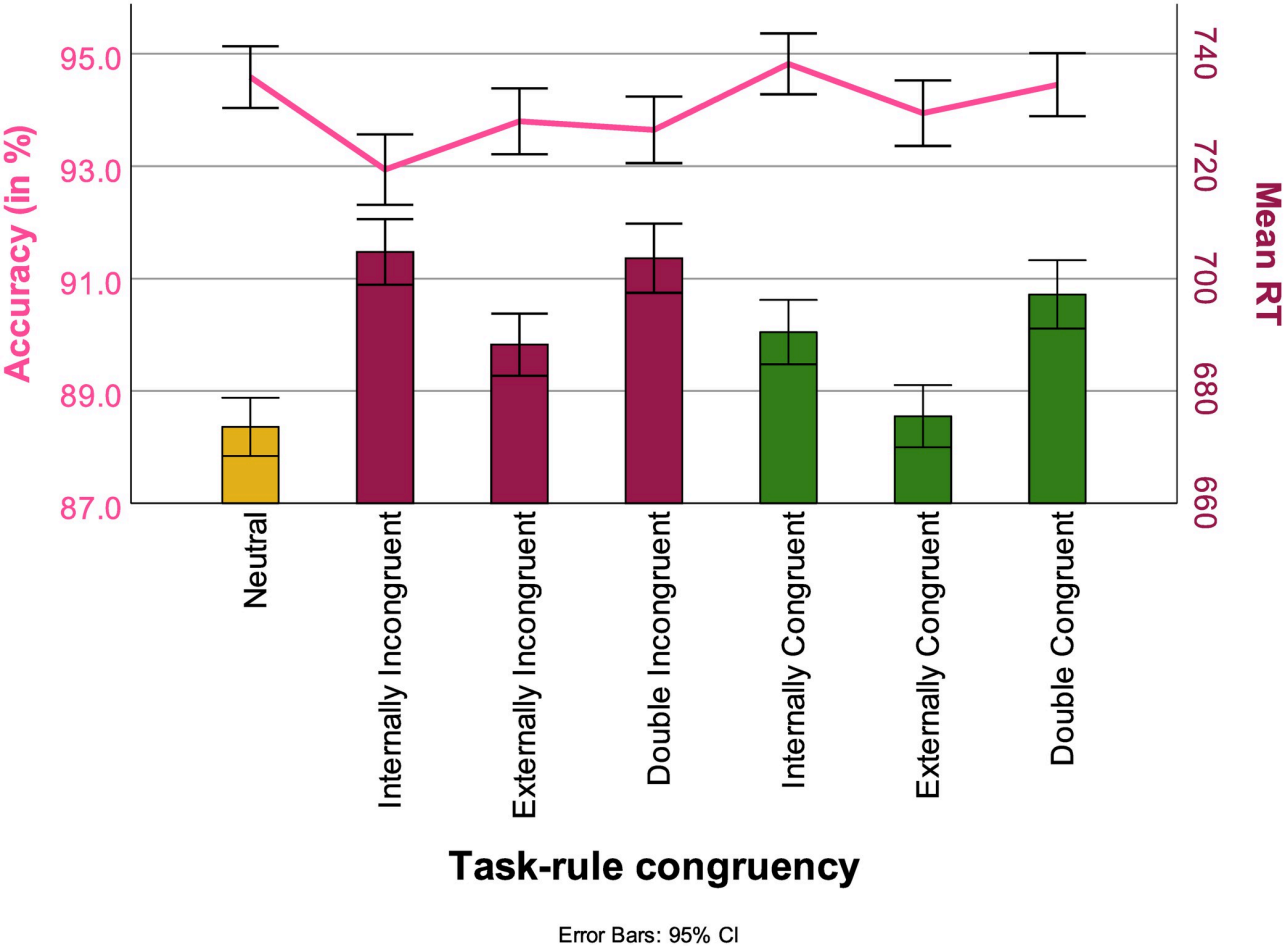
RTs (in ms, bars) and ACC (in %, line) across different types of task-rule congruency, with 95% confidence interval.

RT was also influenced by task-rule congruency. Participants reacted fastest to neutral stimuli (679 ms Std. Err = 13). The longest RTs occurred when participants had stimuli that were either double Task-rule congruent or both Internally and Externally task-rule incongruent (708 ms (both), Std. Err = 1). Most pairwise comparisons were significant at *p* > .05 except for Neutral vs Externally task-rule congruent (*t*(43690), *p* = 1), Internally task-rule incongruent vs Double task-rule incongruent (*t*(44110), *p* = 1), Internally task-rule incongruent vs Double task-rule congruent (*t*(44144), *p* =, Externally task-rule incongruent vs Internally task-rule congruent (*t*(43986), *p* = 1), Externally task-rule incongruent vs Double task-rule congruent (*t*(44199), *p* > 0.1), Double task-rule congruent vs Double task-rule incongruent (*t*(44186), *p* = 1). The bars in Fig 10 represent RT as influenced by task-rule congruency.

### 3.3 ACC

The mixed regression revealed several significant effects, with task-rule congruency, block, task, and switch on the correct or incorrect response dependent variable. The covariance structure chosen for the analysis was ID, as it presented a significantly better model than AR1 (*X*^2^(1)>25, *p* > .001). Except for block, all main effects were significant (Switch *F*(45123, 2) = 40.07, *p* < .001, Task *F*(45123, 3) = 154.48, *p* < .001, task-rule congruency *F*(45123, 6) = 4.9, *p* < .001, Block *F*(45123, 4) = 1.299*p* > .3).

#### 3.3.1 Switches

As expected, repetition trials were the most accurate (95,1%, Std. Err. = 0.3%) (Repeat vs Internal switch *t*(45123), *p* < .001, Repeat vs external switch *t*(45123), *p* < .001). Other than with RTs, Internal switch trials were the least accurate (92.8% ms, Std Err. = 0.3%) than External switch trials (93.5% ms, Std Err. = 0.3%) (Internal vs external switch *t*(45123), *p* < .05). Accuracies across the types of switches are represented by the line in Fig 7.

#### 3.3.2 Tasks

In terms of ACC, most tasks differed from each other significantly. Participants were most accurate in the visual tasks (Cold/Hot 95.8%, Vertical/ Horizontal 94.9%) compared to the numeric tasks (Low/High 94.7%, Even/Odd 89.9%). The only tasks that did not differ from each other significantly were Low/High and Vertical/ Horizontal (Low/High vs Vertical/Horizontal *t*(42557), *p* = 1.0). Accuracies across tasks are represented by the line in Fig 8.

#### 3.3.3 Block

The main effect of block was not significant (*F*(14052, 4) = 12.37*p* > .2). Accuracies across blocks are represented by the line in Fig 9.

#### 3.3.4 Task-rule congruency

ACC was also influenced by congruency. Responses were most accurate when presented with internally task-rule congruent stimuli (94.6% Std. Err = 1.1%). Post-hoc tests showed that only internally task-rule incongruent trials resulted in a significant decrease in performance compared to neutral trials (92.8% Std. Err = 1.1%; *t*(45123), *p* < 0.001). Accuracies dependent on task-rule congruency are represented by the bars in Fig 10.

### 3.5 Exploratory analysis

#### 3.5.1 Rebound effects

**Fig 11:**
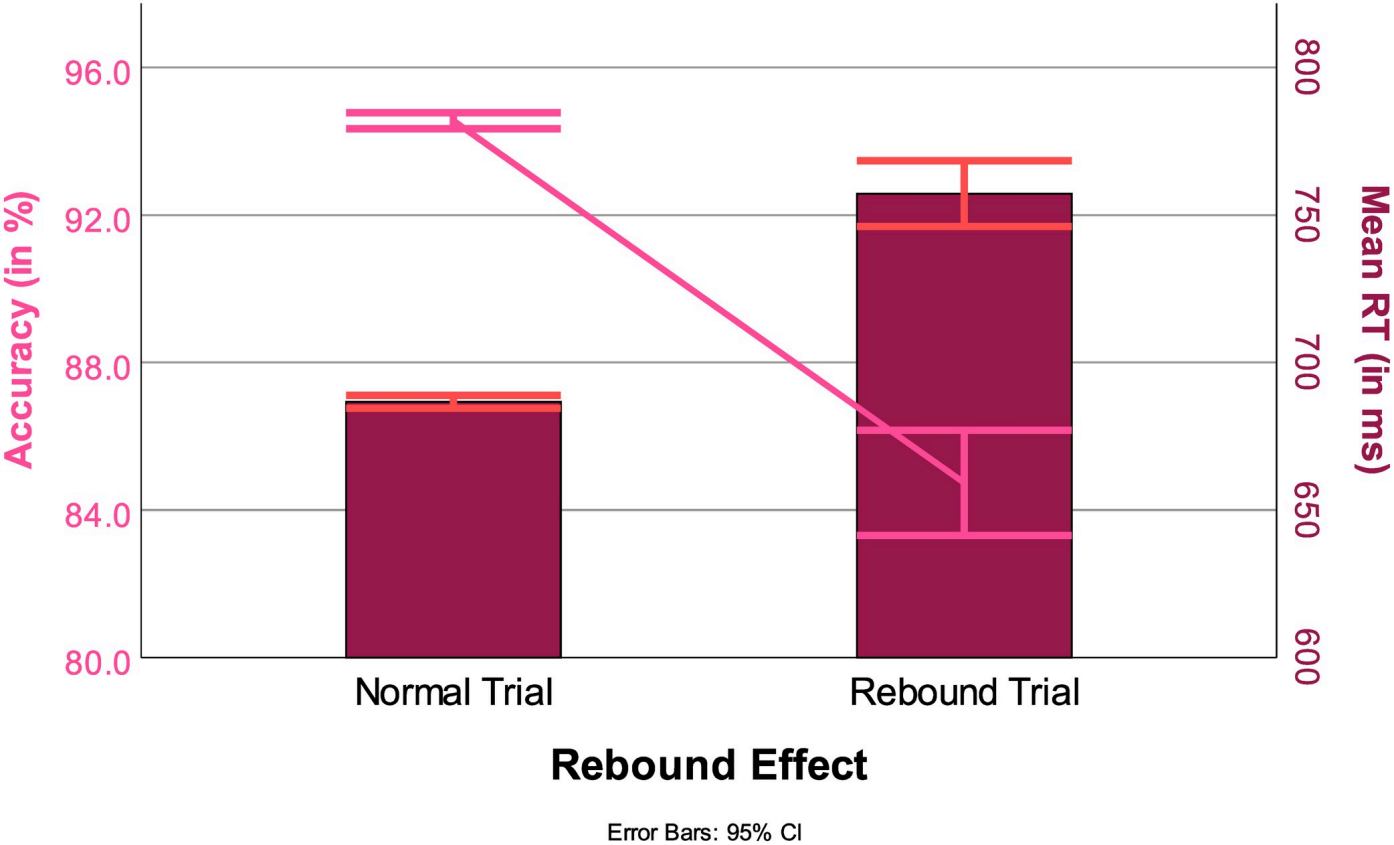
RTs (in ms, bars) and ACC (in %, line) for the rebound effect, with 95% confidence interval.

The Mann-Whitney U tests on RT and ACC for trials following an error were both significant (RT: *z* = 14.66, *p* < 0.001, ACC *z* = −19.98, *p* < 0.001). Following an error, participants were 71 ms slower, with the likelihood of making an error increasing by almost 10% (Fig 11).

#### 3.5.2 Task-rule-congruent convergence

**Fig 12:**
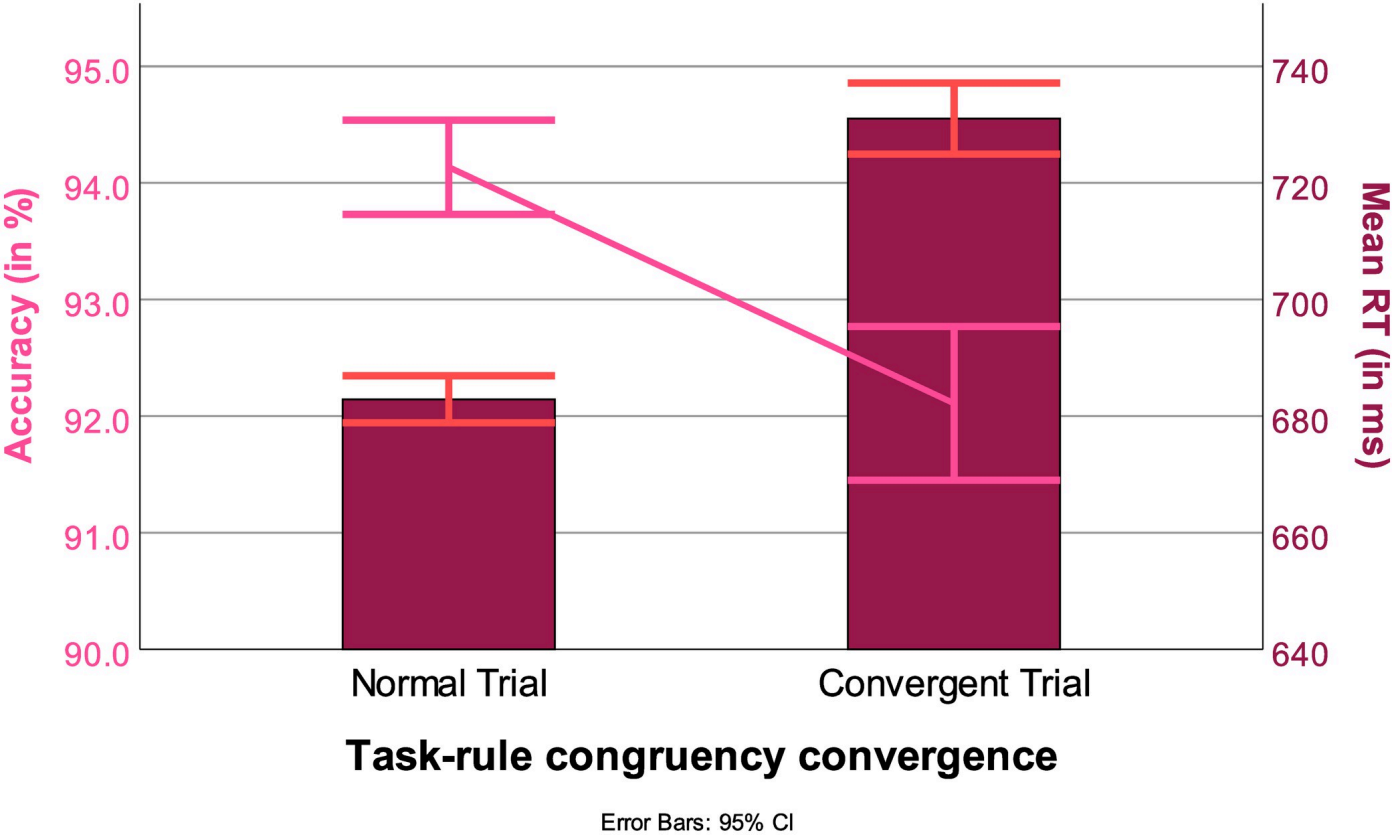
RT (in ms, bars) and ACC (in %, line) for the effect of congruency task convergence, with 95% confidence interval.

The Mann-Whitney U tests on RT and ACC for trials in which the task-rule incongruent information that was presented was coherent with the task of the previous trial were both significant (RT: *z* = 14.50*p* > 0.001, ACC *z* = −5.73, *p* < 0.001). The task-rule congruency convergent trials resulted in an increase of RT of 48 ms and an increased likelihood of making an error by 2%. Results are summarized in Fig 12.

#### 3.5.3 Timepoint mixed model

**Fig 13:**
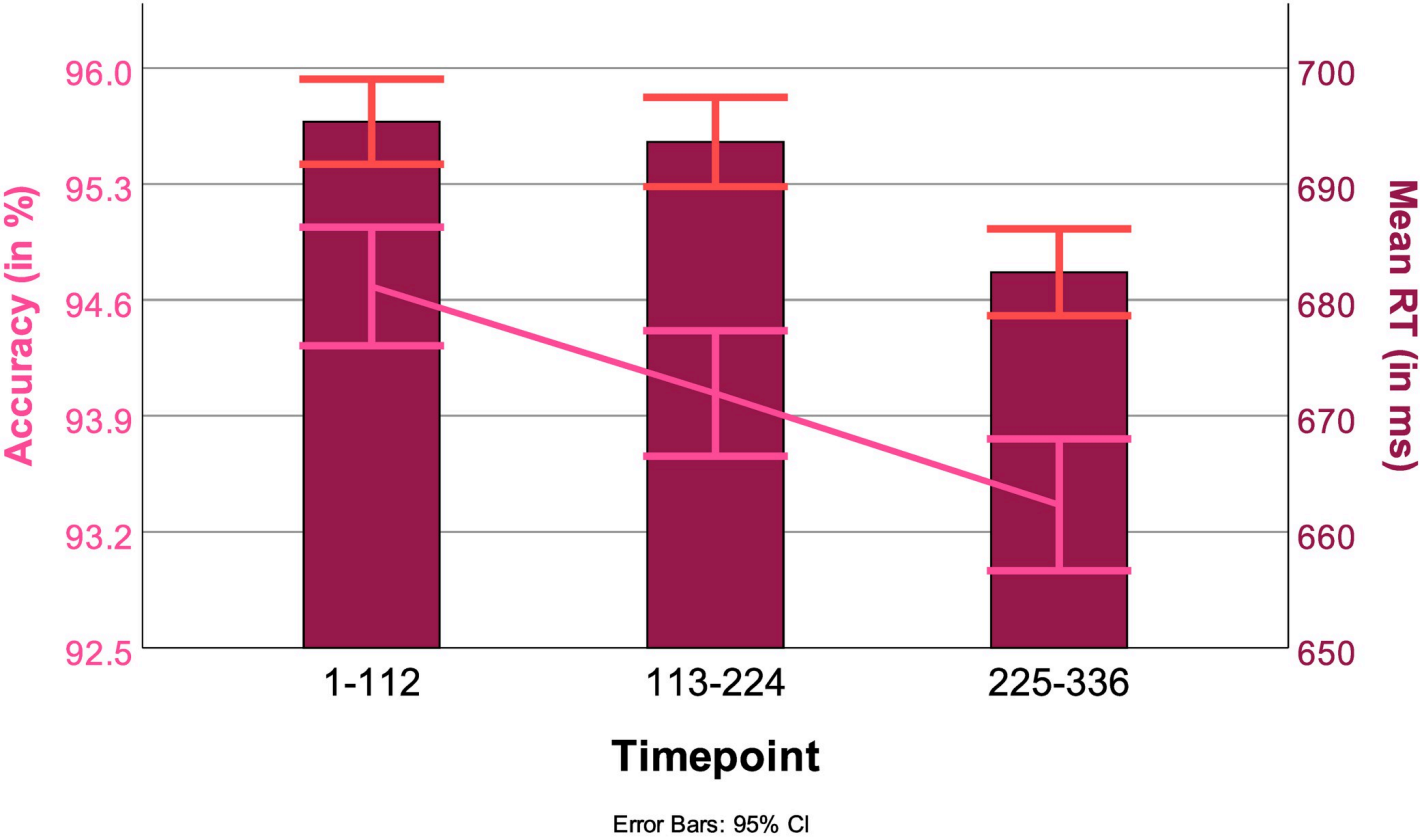
RTs (in ms, bars) and ACC (in%, line) across time points, with 95% confidence interval.

The effect of timepoint on RT was significant (Timepoint: *F*(45123, 2) = 4.83*p* < .01) in the analysis as well as its interaction with both the factors Switch and Block (Switch: *F*(45124, 4) = 2.79, *p* < .05, Block: *F*(45123, 8) = 4.20, *p* < .001). Pairwise comparisons between time points showed a significant difference between the first and the last time point (1–112 vs 225–336: *t*(45123), *p* < 0.01) and the second and third time points (113–224 vs225–336: *t*(45123), *p* < 0.05). RTs decreased across time points. With regard to ACC the main effect of timepoint was significant (Timepoint: *F*(45123, 2) = .06, *p* < .01). The interaction between task and time point was also significant (Task & Timepint: *F*(45123, 6) = 3.09, *p* < .01). Pairwise comparisons between timepoints showed a significant decrease (94.4% to 93.4%) in ACC from timepoint 1 to timepoint 3 (113–224 vs 225–336: *t*(45123), *p* < 0.01). Results are summarized in Fig 13.

#### 3.5.4 Simplified task-rule congruency models

**Fig 14:**
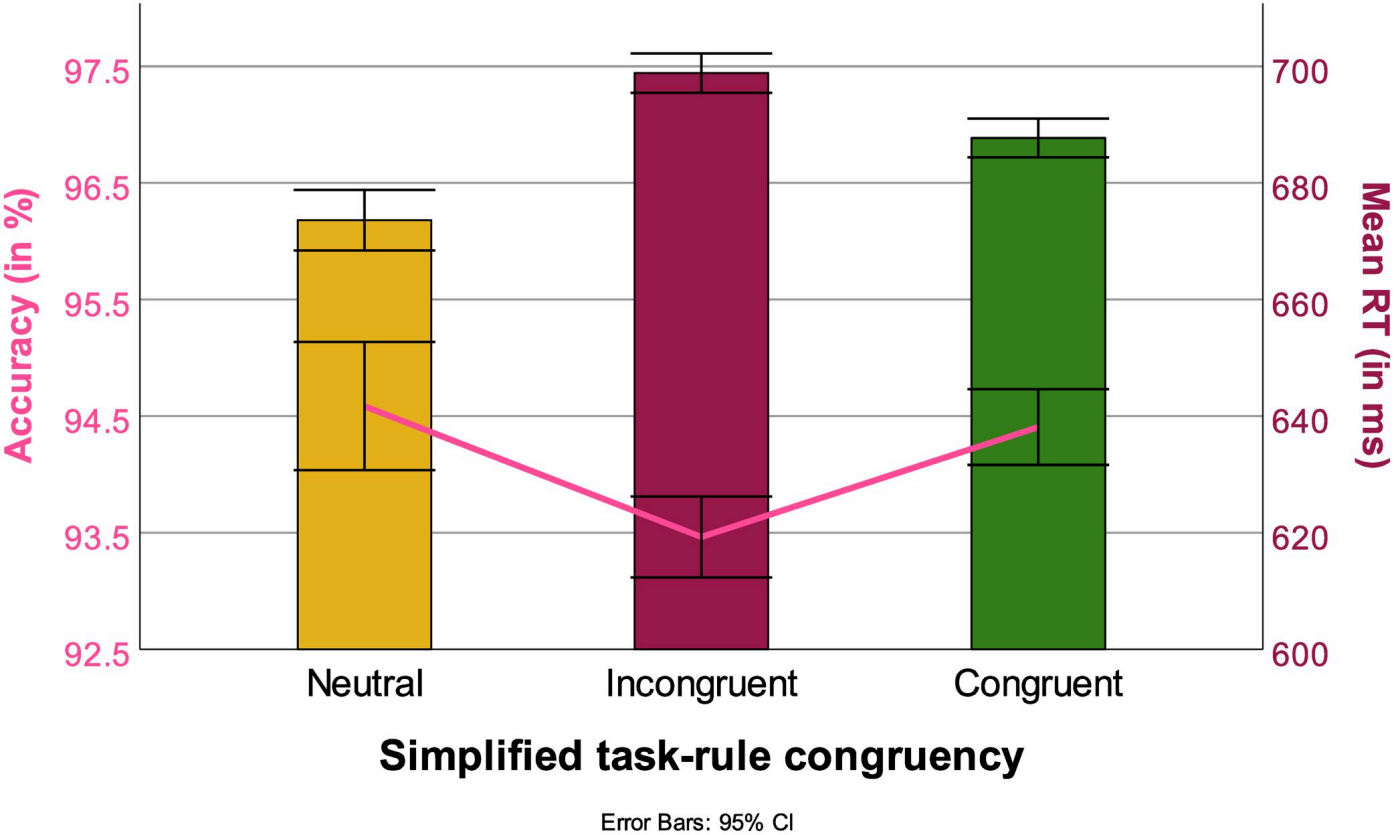
RTs (in ms, bars) and ACC (in %, line) for each simplified task-rule congruency condition, with 95% confidence interval.

The simple task-rule congruency was a significant factor in the mixed model (simplified task-rule congruency: *F*(300041, 2) = 32.23, *p* < .001) with RT as a dependent variable. The Interactions between simplified task-rule congruency and Switch, Block as well as Task were also significant (Switch: *F*(30041, 8) = 3.83, *p* < .005, Block: *F*(30041, 8) = 4.78, *p* < .001, Task: *F*(30041, 6) = 5.65, *p* < .001). All pairwise comparisons were significant. Task-rule neutral trials (679 ms) were the fastest followed by task-rule congruent (693 ms) and finally task-rule incongruent trials (702 ms) (task-rule neutral vs task-rule congruent: *t*(45123), *p* < .001, task-rule neutral vs task-rule incongruent *t*(45123), *p* < .001, task-rule congruent vs task-rule incongruent: *t*(45123), *p* < .001). A similar main effect was also found in the analysis with ACC as a dependent variable (simplified task-rule congruency: *F*(45123, 2) = 9.46, *p* < .001). No interactions were significant. The pairwise comparisons showed a significant difference between task-rule incongruent trials with both task-rule neutral and task-rule congruent trials (task-rule neutral vs task-rule congruent: *t*(45123), *p* < .005, task-rule congruent vs task-rule incongruent: *t*(45123), *p* < .001). However, task-rule congruent and task-rule neutral trials did not differ significantly (task-rule neutral vs task-rule incongruent *t*(45123), *p* = 1). Results are summarized in Fig 14.

#### 3.5.5 Questionnaires

**Fig 15:**
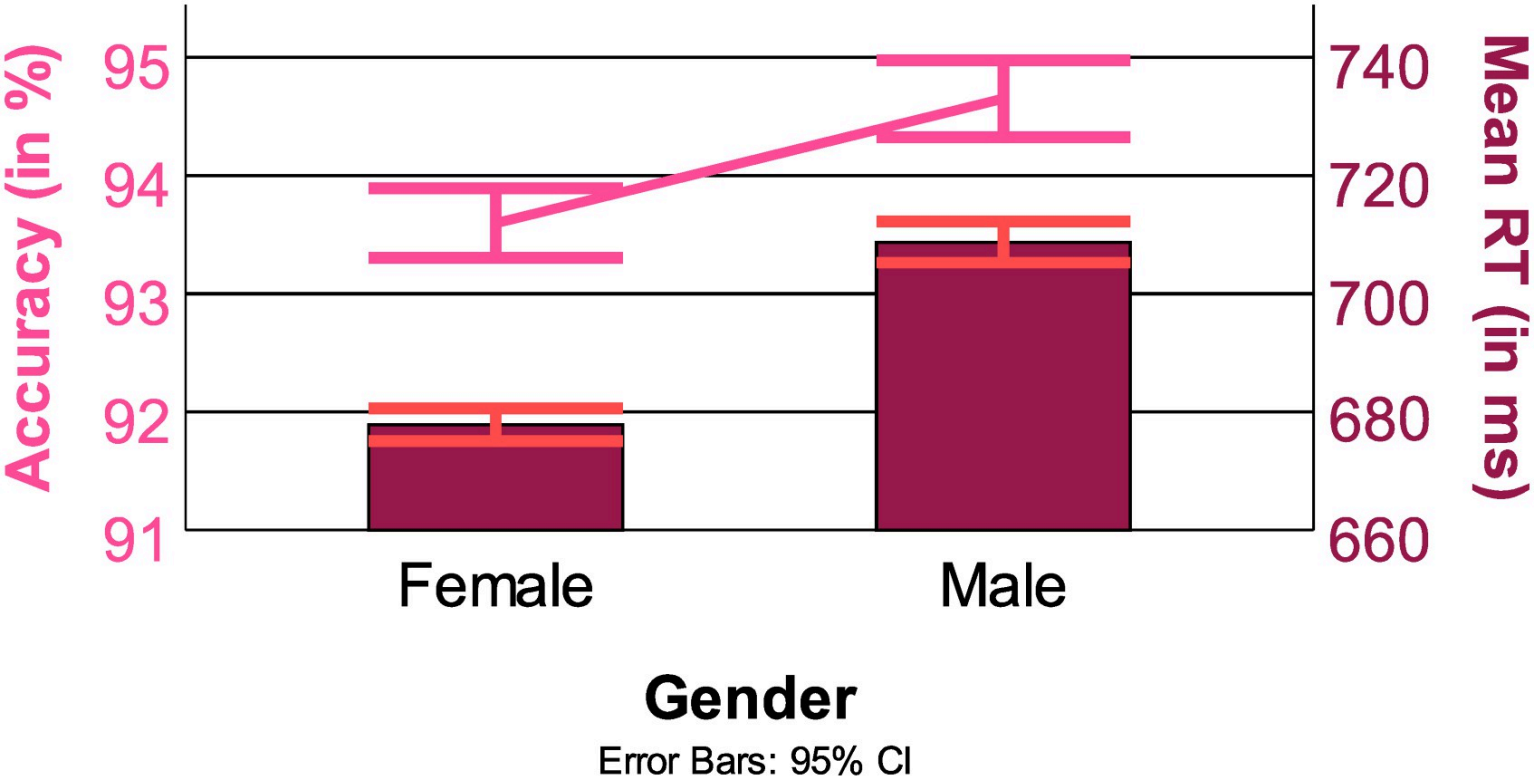
Summary of effects of gender on RT (in ms, bars) and ACC (in %, line), with 95% confidence interval.

The Mann-Whitney U tests of gender on RT and ACC were both significant (RT: *z* = 14.00, *p* < 0.001, ACC *z* = 4.64, *p* < 0.001, see Fig 15 Top). Female participants were less accurate but responded faster.

As expected, the repeated measures ANOVA showed an increase in SPS scores from pre-task measurement to post-task measurement only during the testing session (*F*(119, 1) = 13.20, *p* < .001, *t*(119) = −5.430, *p* < .001).

As with the SPS, as expected, the repeated measures ANOVA showed that reported fatigue increased from pre-task measurement to post-task measurement only during the testing session. (*F*(119, 1) = 17.88, *p* < .001, *t*(119) = −6.121, *p* < .001).

## 4 Discussion

Cognitive Flexibility is an essential factor in complex operations [21, 34–36]. While much fundamental research has explored this issue [1, 3–5, 7, 10, 15], there has not yet been a large shift toward more ecological tasks. One significant difference between real Task-Switch protocols and ecological applications is that fundamental tasks are similar while real-world applications may require operators or persons to switch between very dissimilar tasks. It was the goal of this experiment to investigate to what extent similarity between tasks influences Task-Switch costs. In addition, a particular focus was laid on aspects of mental fatigue.

The protocol required participants to switch between 2 pairs of two tasks, which within pairs were more similar and more dissimilar between pairs. RTs, as well as accuracies, were recorded.

The KSS and SPS questionnaires showed increased mental fatigue on a subjective scale following the testing session but not following the training session.

Contrary to our expectations, the participants’ performance did not decrease significantly across blocks. RTs improved significantly. This is most likely attributable to a flaw in the protocol by not adequately accounting for learning effects. While any observable trend in the decrease of ACC between blocks was non-significant, this was not the case for the within-block (timepoint) analysis. Through a single block, participants tended to make increasingly more errors. This supports the notion that mental fatigue was induced during the task. The most likely reason this did not extend between blocks is the recovery period between blocks.

The main interest of this study was to investigate the effect of similarity on cognitive flexibility. The results indicate that Task-Switch costs (both RT and ACC) were significantly impacted by similarity. It would have been expected that trials with the most errors would also have the largest RTs. This was not the case. Participants struggled not to make errors during internal switches (between similar tasks) but could do so rapidly. External switches (between tasks) required the longest RTs but were more accurate. A possible explanation may be that participants used a compensation strategy during the external tasks to reduce errors. However, the task instructions did not explicitly state that ACC was more important than RT. Furthermore, if this were the case, one would expect accuracies to be the same during similar and dissimilar switches. Finally, participants were unaware that the design considered internal or external switches to be two different classes, further undermining the idea of a compensatory strategy. An alternative explanation is linked to the interference and the reconfiguration view.

The reconfiguration theory assumes that relevant task-relevant elements/ representations of the task set must be retrieved and activated before a new task can be performed [11]. If the same task is repeated, the activated elements and representations remain active, no reconfiguration occurs, and responses are faster. In the case of similar switches, some elements/ representations may be capable of remaining activated, resulting in faster RTs. For example, participants need not pay attention to the numeric values in the visual tasks but in both numeric tasks. So when reconfiguring across dissimilar tasks, more elements must be retrieved/ deactivated.

On the other hand, the interference view would assume that dissimilar trials are easier since there is more interference between similar tasks. Perhaps the presented results here show that the reconfiguration view applies better to measures of RT, while the interference view is more applicable to measures of ACC. Besides the results here supporting this argument, there is some intuitive sense to it. Reconfiguration takes time, but interference is what causes errors.

Besides similarity and mental fatigue, conflicting information presented during trials was also investigated during this study. As expected, the fastest RTs were recorded when only relevant information (neutral trials) was given. Neutral trials were not significantly different regarding ACC from the most accurate trials (internally task-rule congruent). While both task-rule congruent and task-rule incongruent trials increased the RTs, the effect was much more pronounced when the trial was task-rule incongruent. A possible explanation for the increase in RTs in congruent trials may be that participants struggled to identify relevant stimuli when presented with multiple bits of information. The differences between internal, external, and double trials were similar for task-rule congruent and task-rule incongruent conditions. In both cases, external information interfered the least (fastest RTs) with the participant’s decisions. Concerning ACC, especially internal task-rule incongruent information seemed to increase errors. Differences between tasks were also observed. Low/ High & Odd/ Even numeric tasks were significantly more difficult for participants than the visual tasks (vertical/ horizontal & cold/ hot). Regarding both ACC and RTs, Even/ Odd was the most difficult, while Cold/ Hot was the most straightforward task. The balanced design of the experiment, as well as the inclusion of this factor in all analyses, mitigates any potential bias on other analyses.

The exploratory analysis of the task-rule congruency convergence shows a novel effect. Suppose a participant is presented with a task and task-rule incongruent information. In that case, that information has a significantly higher impact on the performance if that incongruent information is coherent with the task the participant had to perform in the previous trial. The results show a significantly higher RT and a 2% increase in error rate. This finding results from an exploratory analysis; therefore, the validity is limited. Further studies should test whether this effect is reproducible. To the best of our knowledge, this effect has, in this form, not been observed or at least not been documented before.

The argument could be made that the observed effect is a case of the congruency-frequency effect observed by [48]. The researchers showed that congruency effects were larger if the presented incongruent stimulus had previously occurred more frequently. Our observation differs in two significant ways. The frequency of all stimuli is constant across blocks. Furthermore, the effect is observed across all stimuli of one task, highlighting that the effect is dependent on the task set and not the stimulus.

These findings may be interpreted in two ways. The increase in TSC may be attributable to confusion among the participants as they believe they must repeat the previous task. The results may also support the interference view of cognitive flexibility. Stimuli relevant to the last task reinforce the activation of the previous task set.

Another surprising effect found in the post hoc analysis was the strength of the rebound effect. Participants were around twice as likely to commit another error following an error. Interestingly, this supports the notion that participants were very aware when making errors, as they received no feedback following each trial. Rebound effects are a well-documented phenomenon. For further information, please refer to [47].

This study’s results support that neither the interference nor the reconfiguration views are adequate for explaining Task-Switch Costs and cognitive flexibility.

The reconfiguration view explains why similar tasks would result in faster responses but would also expect lower accuracies when participants switch to dissimilar tasks. The interference view would expect higher RTs and more errors in similar tasks, as a previous trial may interfere with the current one. The suggestion has been made previously that the components to be retrieved depend on the pair of tasks and impact TSC [49, 50] Suppose, however, we assume that both theories are partially true. How could a combined interference and reconfiguration view explain the difference in switch costs between trial types? In a similar switch, some components of the task set remain the same. So, reconfiguration does not change these components, which may positively impact the RT. However, the instructions from the previous trial may still interfere with the current trial, slightly bias the decision-making process, and result in a higher likelihood of error. Monsell et al. [50] made similar arguments in combining the interference and reconfiguration views. Their argument is based on residual switch costs, an increased RT when switching even if participants are given abundant time to prepare for a switch. According to [50] these costs may be explained by interference.

But these results also show the added value of moving towards more ecological experiments. Moving from similar to dissimilar tasks has a different impact on performance. Future studies should test if this effect is only binary or if the distance between two tasks has a linear influence on performance. Congruency Task Convergence is a fascinating effect that deserves closer attention. A study dedicated to it must first reproduce it reliably to validate its occurrence. The here presented results may also be helpful outside of the academic world. The understanding that similarity between tasks is a source of error is a powerful piece of information that can be used to create interfaces and working procedures that account for or mitigate said effects.

Several limitations have to be taken into account when interpreting the results of this study. The effects of mental fatigue did not accumulate across blocks as expected. In addition to learning effects likely to have had the most substantial impact on performance, compensatory strategies may as well have influenced the performance. While the link between mental fatigue and performance is well-established, it is not uncommon to not detect the expected behavioural change over time [51]. Maintaining or improving on performance may also be a factor of motivation that can overpower mental fatigue. It may also be an increase in effort as a response to mental fatigue that negates the decrement in performance.

The significant differences in performance between tasks present no limitation to the results presented here as the design was balanced, and the factor was considered during the statistical analysis.

Lastly, it should be noted that following the data collection, a minor bug in the code was detected. In double task-rule congruent or double task-rule incongruent trials, the same external task was presented in each of these trials from block two on. However, post hoc comparison of these trials in the first and the remaining blocks showed no effect of this configuration on the data. This was tested with a non-parametric test by comparing RTs and accuracies of relevant trials to search for any difference in performance.

In a future study, it would also be of interest the extent to which errors occurred because the response keys were the same for all tasks.

## 5 Conclusion

This study on how task similarity affects cognitive flexibility has yielded several noteworthy findings. As similarity decreases between tasks, participants get slower in switching; similar functions, on the other hand, produce more errors. One interpretation of these findings is that the interference view of cognitive flexibility is better suited to explain changes in error-making. In contrast, the reconfiguration view is more applicable to measures of RT. These findings also show us that findings from fundamental research are not readily applicable to more ecological settings where similarity between tasks is more scarce than in traditional task switch paradigms. To the best of our knowledge, the effect of task-rule congruency convergence has not yet been reported in the literature. If reproducible, this effect would have implications for our understanding of task switch and necessitate adapting future paradigms to account for it. With only two tasks, this effect is not observable. In conclusion, this article promotes a shift toward adopting fundamental paradigms of task switches in ecological settings while proposing novel aspects that shape the understanding of the mechanisms of cognitive flexibility.

## Supporting information

S1 Fig The demographics questionnaire includes questions about age, education, gender and employment. (TIF)

S2 Fig The shortened version of the Edinburgh Handedness Inventory encompasses 4 items (e.g. writing) scored on a 5-point scale ranging from:” Always right; usually right; both equally; usually left; always left”. This shortened version is a faster measure while maintaining its reliability [40].
(TIF)

S3 Fig The Ishihara test for colourblindness [41] encompasses stimuli that can determine the degree and type of colourblindness a person has. It was presented in print, using a validated test version made available by the US Department of Infectious Diseases. Participants were informed that a licensed physician is required for a medically accurate test. Should the suspicion of colourblindness arise during the assessment, participants were notified and advised to seek a medical professional.
(TIF)

S4 Fig The St Mary’s Hospital Sleep Questionnaire was used to estimate the sleep quality on the previous night. A translated version of this questionnaire has been produced [46]. The scoring of this questionnaire is not standardized due to the use of Likert scales and free responses. In addition, two questions regarding caffeine consumption have been added to this questionnaire.
(JPG)

S5 Fig The Samn-Perelli Scale is a 7-point Likert scale used to assess fatigue [42, 45]. (TIF)

S6 Fig The Karolinska Sleepiness Scale (KSS) is a 9-point Likert scale, ranging from “extremely 1 = alert” to “9 = extremely sleepy–fighting sleep” [44]. (TIF)
