# Supplementary figures and images for: Double task switching: An investigation into the effects of similarity and task-rule congruency on cognitive flexibility
Source: PLoS One. 2024 Oct 3;19(10):e0305675. doi: 10.1371/journal.pone.0305675 (PMC11449286; doi:10.1371/journal.pone.0305675)

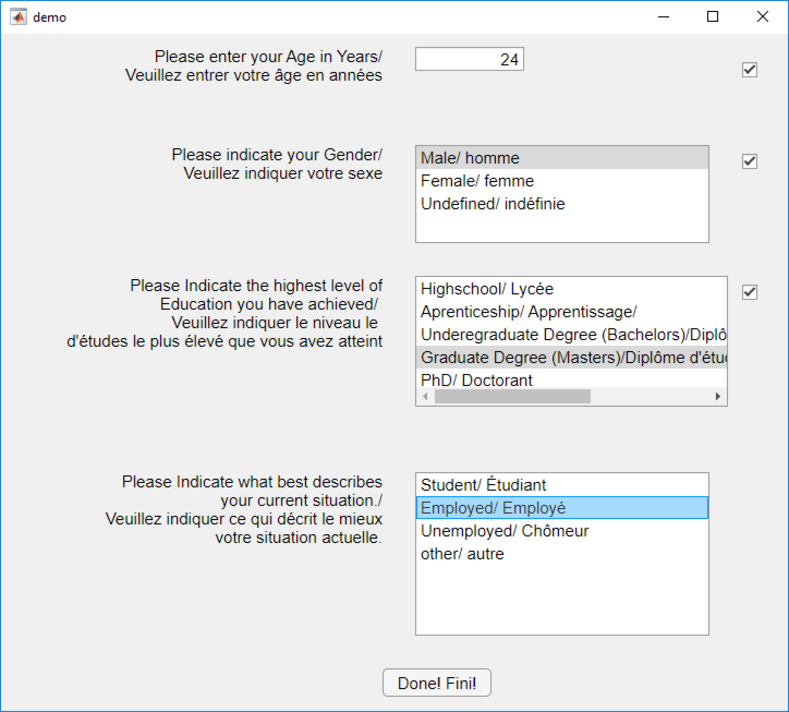

Supplement: S1 Fig — (TIF) [file pone.0305675.s001.tif]

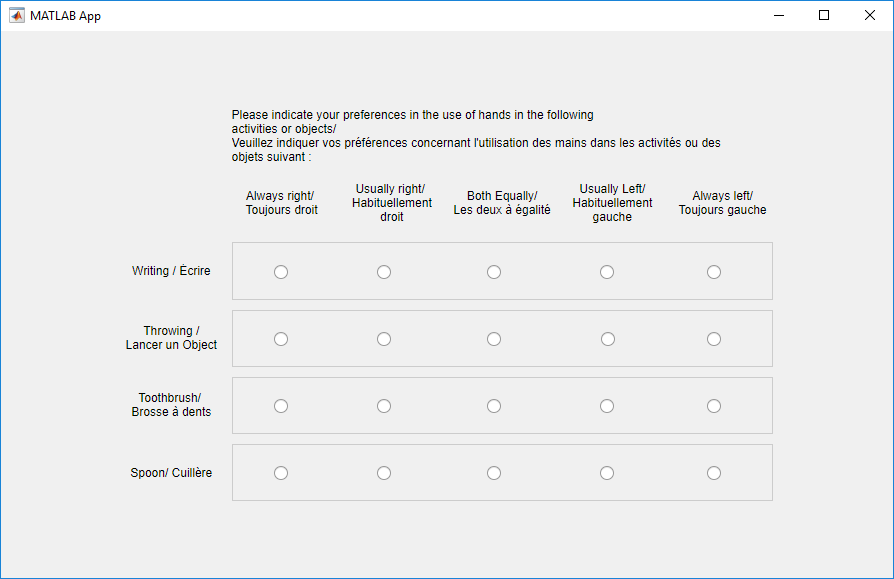

Supplement: S2 Fig — This shortened version is a faster measure while maintaining its reliability [40]. (TIF) [file pone.0305675.s002.tif]

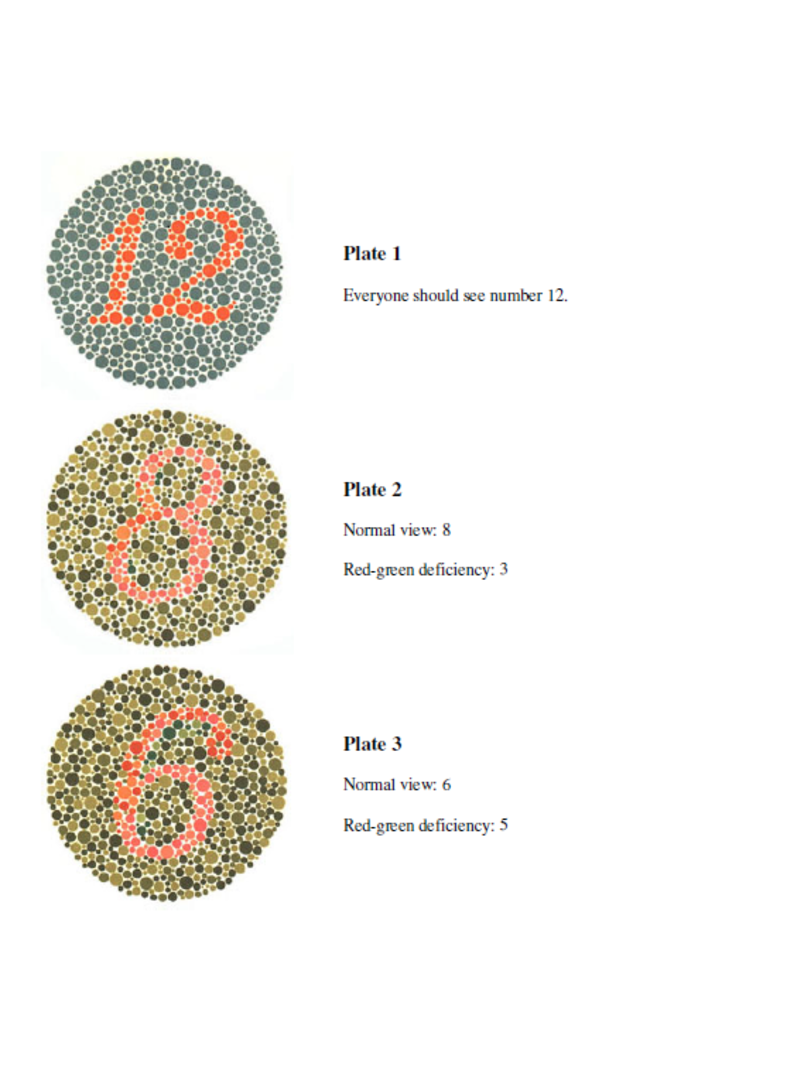

Supplement: S3 Fig — It was presented in print, using a validated test version made available by the US Department of Infectious Diseases. Participants were informed that a licensed physician is required for a medically accurate test. Should the suspicion of colourblindness arise during the assessment, participants were notified and advised to seek a medical professional. (TIF) [file pone.0305675.s003.tif]

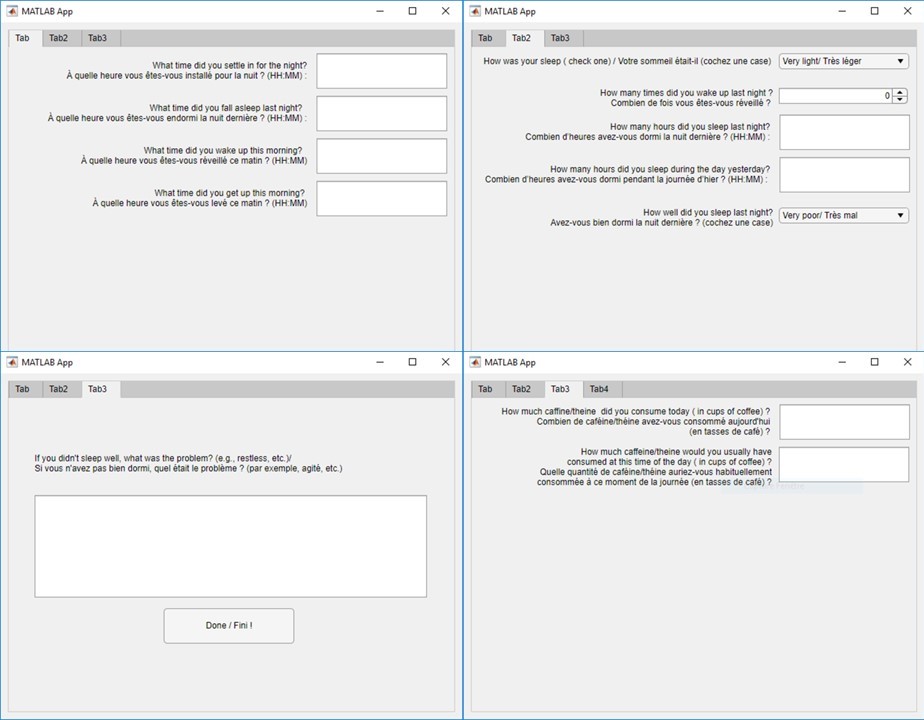

Supplement: S4 Fig — A translated version of this questionnaire has been produced [46]. The scoring of this questionnaire is not standardized due to the use of Likert scales and free responses. In addition, two questions regarding caffeine consumption have been added to this questionnaire. (JPG) [file pone.0305675.s004.jpg]

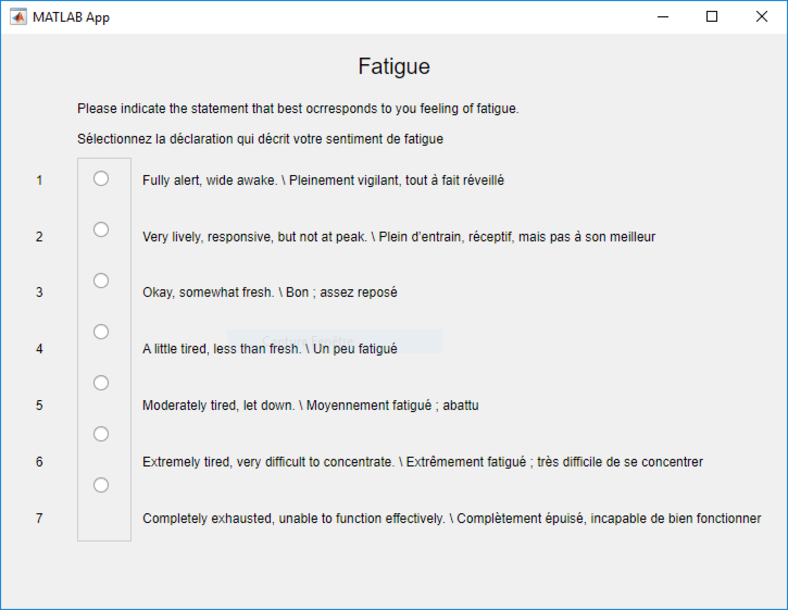

Supplement: S5 Fig — (TIF) [file pone.0305675.s005.tif]

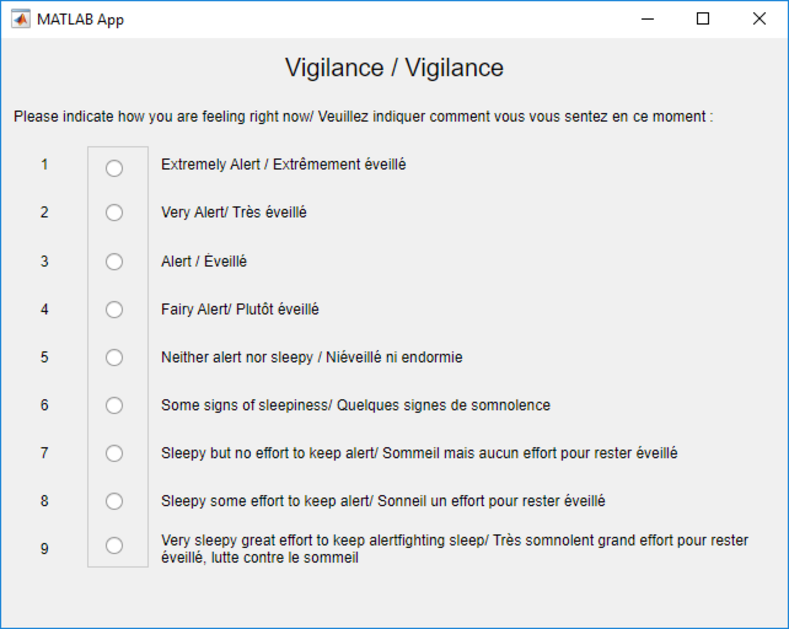

Supplement: S6 Fig — (TIF) [file pone.0305675.s006.tif]
